# Guidance for identification and treatment of individuals with attention deficit/hyperactivity disorder and autism spectrum disorder based upon expert consensus

**DOI:** 10.1186/s12916-020-01585-y

**Published:** 2020-05-25

**Authors:** Susan Young, Jack Hollingdale, Michael Absoud, Patrick Bolton, Polly Branney, William Colley, Emily Craze, Mayuri Dave, Quinton Deeley, Emad Farrag, Gisli Gudjonsson, Peter Hill, Ho-lan Liang, Clodagh Murphy, Peri Mackintosh, Marianna Murin, Fintan O’Regan, Dennis Ougrin, Patricia Rios, Nancy Stover, Eric Taylor, Emma Woodhouse

**Affiliations:** 1 Psychology Services Limited, London, UK; 2 South London & Maudsley NHS Foundation Trust, Service for Complex Autism and Associated Neurodevelopmental Disorders, London, UK; 3 Department of Children’s Neurosciences, Evelina London Children’s Hospital, Guy’s and St Thomas’ NHS Foundation Trust, London, UK; 4 Department of Women & Children’s Health, King’s College, London, UK; 5 Oxford ADHD & Autism Centre, Oxford, UK; 6 CLC Consultancy, Perth, UK; 7 South London & Maudsley NHS Foundation Trust, National Autism Unit, Kent, UK; 8 Positive Behaviour, Learning Disability, Autism and Mental Health Service (PALMS) Hertfordshire Communication Disorders Clinics, Hertfordshire Community NHS Trust, St Albans, UK; 9 South London & Maudsley NHS Foundation Trust, Maudsley Health, Abu Dhabi, UAE; 10 Department of Psychology, Institute of Psychiatry, Psychology and Neuroscience, King’s College London, London, UK; 11 Private Practice, London, UK; 12 Great Ormond Street Hospital, London, UK; 13 Behavioural and Developmental Psychiatry Clinical Academic Group, Behavioural Genetics Clinic, National Adult Autism and ADHD Service, South London and Maudsley Foundation NHS Trust, London, UK; 14 Department of Forensic and Neurodevelopmental Sciences, Institute of Psychiatry, Psychology & Neuroscience, King’s College London, London, UK; 15 Anna Freud Centre, London, UK; 16 Helen Arkell Centre, Guildford, UK; 17 Department of Child and Adolescent Psychiatry, Institute of Psychiatry, Psychology and Neuroscience, King’s College London, London, UK; 18 Diagnostic Assessments and Treatment Services (DATS), Hertfordshire, UK; 19 Stover Consultancy, Santa Barbara, CA USA

**Keywords:** hyperactivity disorder/hyperactivity disorder (ADHD), Autism spectrum disorder (ASD), Comorbidity, Assessment, Treatment, Guidance, Consensus, Interventions, Education, School, Occupation, Psychiatry, Psychology

## Abstract

**Background:**

Individuals with co-occurring hyperactivity disorder/hyperactivity disorder (ADHD) and autism spectrum disorder (ASD) can have complex presentations that may complicate diagnosis and treatment. There are established guidelines with regard to the identification and treatment of ADHD and ASD as independent conditions. However, ADHD and ASD were not formally recognised diagnostically as co-occurring conditions until the Diagnostic and Statistical Manual of Mental Disorders 5 (DSM-5) was published in 2013. Hence, awareness and understanding of both conditions when they co-occur is less established and there is little guidance in the clinical literature. This has led to uncertainty among healthcare practitioners when working with children, young people and adults who present with co-existing ADHD and ASD. The United Kingdom ADHD Partnership (UKAP) therefore convened a meeting of professional experts that aimed to address this gap and reach expert consensus on the topic that will aid healthcare practitioners and allied professionals when working with this complex and vulnerable population.

**Method:**

UK experts from multiple disciplines in the fields of ADHD and ASD convened in London in December 2017. The meeting provided the opportunity to address the complexities of ADHD and ASD as a co-occurring presentation from different perspectives and included presentations, discussion and group work. The authors considered the clinical challenges of working with this complex group of individuals, producing a consensus for a unified approach when working with male and female, children, adolescents and adults with co-occurring ADHD and ASD. This was written up, circulated and endorsed by all authors.

**Results:**

The authors reached a consensus of practical recommendations for working across the lifespan with males and females with ADHD and ASD. Consensus was reached on topics of (1) identification and assessment using rating scales, clinical diagnostic interviews and objective supporting assessments; outcomes of assessment, including standards of clinical reporting; (2) non-pharmacological interventions and care management, including psychoeducation, carer interventions/carer training, behavioural/environmental and Cognitive Behavioural Therapy (CBT) approaches; and multi-agency liaison, including educational interventions, career advice, occupational skills and training, and (3) pharmacological treatments.

**Conclusions:**

The guidance and practice recommendations (Tables 1, 4, 5, 7, 8 and 10) will support healthcare practitioners and allied professionals to meet the needs of this complex group from a multidisciplinary perspective. Further research is needed to enhance our understanding of the diagnosis, treatment and management of individuals presenting with comorbid ADHD and ASD.

## Background

Approximately 5% of the population worldwide is reported to have a diagnosis of hyperactivity disorder/hyperactivity disorder (ADHD) [1], a disorder often associated with co-occurring conditions that can complicate identification and treatment [2]; autism spectrum disorder (ASD) is among those conditions that are commonly found to co-exist with those diagnosed with ADHD at a reported rate (obtained by meta-analysis) of 21% [3].

There are established guidelines regarding the identification and treatment of ADHD and ASD as independent conditions. Indeed, this comorbidity was not formally recognised diagnostically until the fifth revision of the Diagnostic and Statistical Manual of Mental Disorders (DSM-5) [4]; hence, awareness and understanding of them as co-occurring conditions is less established. This has led to uncertainty among healthcare practitioners when working with children, young people and adults who present with both ADHD and ASD. It is further complicated by the expansion of the concept of ASD in recent years, which means that (although ASD is a neurodevelopmental disorder) many people in the spectrum are intellectually and emotionally able and their ‘impairment’ primarily relates to stigmatisation within society (for instance, in being excluded unnecessarily from employment). Nevertheless, mental health needs may arise from co-existing conditions (such as anxiety and ADHD). This consensus therefore included the needs of those who are functionally impaired as well as those who are more broadly considered ‘neurodiverse’.

### hyperactivity disorder/hyperactivity disorder

ADHD is a childhood onset, neurodevelopmental disorder with genetic and environmental origins [5] characterised by pervasive behavioural symptoms of hyperactivity, inattentiveness and impulsivity that have been present for at least 6 months and adversely impact on daily functioning and development [4]. ADHD is highly prevalent. Research combining data from multiple sources and analysed using meta-regression reported a worldwide-pooled estimate of 5.29–7.2% in children [1, 6], 2.5% in adults [7] and between 2.8% in older adulthood [8]. Symptoms must persist across the lifespan, although the relative balance and the specific manifestations of inattentive and hyperactive-impulsive characteristics vary across individuals, and may change over the course of development, e.g. in particular hyperactive-impulsive symptoms commonly reduce with age [9, 10].

The diagnostic criteria for ADHD are presented in DSM-5 [4] and the World Health Organization’s International Statistical Classification of Diseases, 11th edition beta, (ICD-11) [11]. Whilst the basic symptom criteria did not change from the revision of DSM-IV [12], DSM-5 increased the typical age of onset to age 12 (previously age 7). The definition of ADHD was expanded to more accurately characterise the experience of adults, and the number of symptoms was relaxed for adults (> 17 years) who are now required to have five or more symptoms that have persisted for at least 6 months in the inattention and/or hyperactive-impulsive domains. For a diagnosis made in childhood, the number of symptoms remained as 6, respectively. Symptoms must be pervasive and interfere with or reduce the quality of social, academic and occupational functioning. They must not be better explained by other conditions, such as intellectual disability. The change in nomenclature from ‘subtypes’ in DSM-IV to ‘presentations’ in DSM-5 reflects increasing evidence that symptoms are often fluid within individuals across their lifespan rather than stable traits. The ‘presentation’ represents the person’s current symptomatology which may change over time: For example, in ADHD, inattentiveness may be relatively stable across development, but hyperactivity and impulsiveness often wane with age. Also, importantly, ASD is no longer an exclusion criterion, which is a fundamental change from DSM-IV and this, together with other comorbid conditions, should be noted. The advance preview of ICD-11 was published in June 2018, and this no longer refers to ADHD as hyperkinetic disorder. Other than that, it appears to be essentially similar to DSM-5 [13, 14].

Multi-morbidity is common in both children and adults of both sexes. In children, around one-half will have at least one psychiatric disorder comorbid to ADHD and around one-quarter will have two or more comorbid disorders [15]. These are typically ‘current’ episodes of comorbidity, the most frequent in childhood being disruptive behavioural disorders (e.g. oppositional defiant disorder, conduct disorder), anxiety (e.g. generalised anxiety disorder, social anxiety, obsessive-compulsive disorder) and mood (e.g. depression, bipolar disorder). Others include specific developmental disorders of language, learning and motor development, autism spectrum disorders and intellectual disability, many of which present across the lifespan [3, 15–18]. Adults with ADHD also experience high rates of co-occurring conditions, especially anxiety, mood and substance use disorders (including cigarette smoking) [19, 20]. Therefore, the clinical presentation is complex, and this is often further complicated by academic and social impairments, leading to academic and occupational failure, delinquent and antisocial behaviour [20–22].

### Autism spectrum disorders

The worldwide prevalence of ASD is around 1%, but some data suggest it may be higher [23, 24]. Following the publication of DSM-5 in 2013, ASD is now characterised by two categories: (1) persistent deficits in the ability to initiate and to sustain reciprocal social interaction and social communication and (2) restricted, repetitive and inflexible patterns of behaviour, interests or activities, including differences in sensory sensitivities and interests. Individuals with ASD exhibit a range of intellectual and language functioning. Symptoms typically present in early childhood (by 2–3 years of age) with or without language delay and may be associated with early developmental delays and/or loss of social or language skills. However, many people may not receive a diagnosis of ASD until adulthood. This may be due to a variety of factors including lack of awareness of ASD across the lifespan, poor recognition of ASD in females and/or lack of services [25]. DSM-5 notes that symptoms must be associated with clinically significant impairment in personal, family, social, educational, occupational or other important areas of functioning. Symptoms are usually a pervasive feature of the individual’s functioning which are observable across settings. However, they may vary according to social, educational or other contexts and may be masked by ‘camouflaging’. Symptoms are relatively stable across the lifespan [26, 27], although there may be some developmental gains in adolescence and adulthood due to increased interest in social interaction, behavioural improvement associated with interventions and/or compensations applied and/or the environmental supports provided.

The DSM-5 made substantial amendments to the diagnostic criteria of ASD from previous publications, the most striking being the removal of Asperger’s disorder and pervasive developmental disorder ‘not otherwise specified’ as distinct subtypes and these are now subsumed under the general diagnosis of a scalable severity of ASD. The latter is introduced by a hierarchical classification of the level of support required by the individual, i.e. level 1 ‘requiring support’, level 2 ‘requiring substantial support’ and level 3 ‘requiring very substantial support’. Severity of social communication difficulties and restricted, repetitive behaviours are rated separately, with the recognition that severity may vary by context and fluctuate over time. Additional specifiers should be included recording (a) with or without accompanying intellectual impairment (intellectual disability and ASD frequently co-occur, but symptoms should not be *better* explained by intellectual disability or global developmental delay); (b) with or without language impairment (assessed and described by the current level of verbal functioning, e.g. no intelligible speech, single words only, phrase speech, full sentences, fluent speech). Both receptive and expressive language is considered separately since the former may be relatively delayed in ASD; (c) whether ASD is associated with a known medical or genetic condition or environmental factor; (d) whether ASD is associated with another neurodevelopmental, mental or behavioural disorder; and (e) whether ASD is associated with catatonia.

Hence, DSM-5 has moved away from a polarised distinction of ‘high’ and ‘low’ ASD functioning and instead focuses on whether the condition is associated with or without accompanying intellectual impairments. Severity is classified by the level of support required by an individual, ranging across three levels. DSM-5 further separated out ASD from social communication disorder (SCD); impairments in social communication without the presence of repetitive, restricted or stereotyped behaviours may meet criteria for SCD.

At the time of writing, the ICD-10 is under revision and a detailed version of ICD-11 has yet to be published [28]. It is anticipated that ICD-11 will align with DSM-5, in that ASD can occur with other neurodevelopmental and mental health conditions (including ADHD) if full diagnostic criteria are met. Moreover, Asperger’s syndrome and other generalised developmental disorders will be included within the broader classification of autism and the diagnostic criteria will be organised under the same two categories presented in DSM-5, i.e. (1) difficulties in interaction/social communication and (2) repetitive behaviours (although it seems that ICD-11 will not include sensory sensitivities). Hence, the characteristic specifically relating to language problems has been removed, although ICD-11 seems to consider the loss of previously acquired competencies (such as language, self-care, toileting, motor skills) as a characteristic that should be considered when making a diagnosis. Further, ICD-11 will place less emphasis on the type of play children partake in due to variations across countries and culture but instead focus more of whether children follow or impose strict rules when they play.

ASD co-occurs with several neurodevelopmental, psychiatric and neurological conditions [29, 30]. Indeed, 70% of children with ASD will have at least one co-occurring psychiatric condition and 41% will have two or more, the most common being social anxiety, ADHD and oppositional defiant disorder. Adults also experience high rates of comorbidity, the most common being mood disorders, anxiety disorder and ADHD [31]. Patterns of comorbidity are similar across the lifecycle, however, with individuals presenting with a range of anxiety and mood disorders, including panic attacks, generalised anxiety disorder, obsessive-compulsive disorder and unipolar and bipolar disorders. Hence, as for people with ADHD, the presentation of people with ASD may be complex from both a clinical and behavioural perspective but outcomes for those with ASD are often more marked in terms of functional impairments. Many adults with ASD live independently in adulthood but describe difficulties across a variety of educational and occupational settings and require support (formal or otherwise) from family or professionals [32].

### Co-occurring ADHD and ASD

Previous publications of the DSM prohibited clinicians from making a concurrent ADHD and ASD diagnosis. The change in diagnostic approach taken by DSM-5 most likely reflects a growing body of research over the past decade that has identified both shared and distinct risk factors and phenotypic manifestations, including:

- Many copy number variants (CNVs) and chromosome abnormalities confer risks for ADHD and ASD [33]. A genome-wide association study across five major psychiatric disorders including ADHD and ASD found evidence of shared genetic risk factors between ADHD and ASD, and common molecular pathways and functional domains that are affected by the disorders [34, 35].
- Both conditions frequently co-occur and cluster in families. Multiple interacting genetic factors and their interplay with environmental factors constitute the main causative determinants of both ADHD and ASD [36]. This interaction can affect epigenetic expression such as DNA methylation, histone modification and microRNA expression leading to altered development.
- Brain structure and functional imaging studies show both shared and distinct neuronal features and activities [37]. For example, functional brain magnetic resonance imaging (fMRI) studies report both disorder-specific differences and shared deficits in functional brain activation and behavioural performance in tasks of sustained attention [38] and temporal discounting [39] in youth with ADHD and ASD. The largest structural brain MRI investigation to date of young people and adults with ADHD and ASD notes maturational differences, with ADHD-specific smaller intracranial volume in children and adolescents and ASD-specific thicker frontal cortices in adults [40].
- Both disorders have a male predominance [2, 38]. Although some specific distinctions are present between the two conditions, generally both are commonly associated with social difficulties, executive dysfunction and language and cognitive delays [36, 41–43].

Rates of co-occurrence vary. In children, clinical studies investigating ADHD symptoms in ASD report a co-occurrence ranging between 53 and 78% [44, 45]; whereas in community samples, it is lower at 28–31% [30, 46]. A meta-analysis of the co-occurrence of ASD symptoms in young people from both clinical and community ADHD samples found a comorbid rate of 21% [3]. This suggests that ADHD symptoms may be more likely to be found in people with ASD than vice versa and a meta-analysis of rates of ASD in people with ADHD employing a similar methodology as Hollingdale and colleagues [3] would be helpful to consolidate the available data.

The co-occurrence of ADHD and ASD presents in those both with and without intellectual disability. In a personal communication from Dr. Bhathika Perera, Chair of the Royal College of Psychiatrists’ ADHD in Intellectual Disability Group (a sub-group of their Neurodevelopmental Disorder Special Interest Group), we were informed of a recent UK national audit (in preparation) that showed 70% of people with intellectual disability and ADHD also have autism. When working with this group, however, one needs to be mindful that there may be a difference in conceptualising ADHD in mildly intellectually disabled, verbal ASD individuals in contrast to those who are non-verbal and severely intellectually disabled. This has implications for treatment; for example, agitated states in severely intellectually disabled non-verbal autistic individuals may be confused with hyperactivity, leading to the underlying core problem not being understood and addressed. In turn, this may lead to greater risk of deterioration in the individual’s clinical presentation and associated functioning.

As discussed, comorbidity is very common in both conditions but for those in whom it co-occurs, this may be considerably more marked. Research from a community sample by Simonoff and colleagues [30] found that when ASD was comorbid with ADHD, the risk of receiving a further comorbid diagnosis increased by 14% (from 70% for the ASD group to 84% for the co-occurrence of ASD and ADHD). A prison study of 390 male inmates with ADHD reported that 21.9% screened positive for ASD, and this group with co-occurring ADHD and ASD was at higher risk of developing comorbid psychiatric symptoms than inmates in either group alone [47]. Thus, the co-occurrence of both conditions may increase the risk of developing further psychiatric conditions; indeed, persisting states of anxiety and dysphoria are common in ASD (alone or with comorbid ADHD) and risk of suicide in this group is a concern [48, 49]. However, many individuals do not progress to a level of emotional ‘disorder’ that persists for a substantial period but instead experience mood instability, characterised by extreme fluctuations in mood that change rapidly over the course of a day [50]. This may be perceived by healthcare practitioners as less debilitating and of lower concern, but for the individual (and their families/carers), these extreme fluctuating emotional states are exhausting and deeply distressing.

There are diagnostic challenges for both conditions especially for those with subtle or ‘mild’ presentations, when difficulties are ‘masked’ by other comorbid conditions, ‘camouflaged’ by compensatory strategies, and/or when there is limited information about childhood functioning when making the diagnosis of ADHD or ASD for the first time in adults. Other challenges relate to teasing out symptoms and difficulties that may be adaptive in certain situations or settings. For example, an individual may be friendly and socially proactive, but the quality of these interactions may be gauche or ‘odd’ in nature. Concentration problems and/or overactivity may be less evident when individuals are engrossed in a topic or activity of special interest. Distraction, in terms of switching tasks by moving from one incomplete activity to another, may also be influenced by the level of interest in the task and/or modified by a tendency to become preoccupied with the task or due to a resistance to change. There are also diagnostic challenges when working with individuals at the other end of the spectrum, i.e. when assessing ASD in severely/profoundly disabled children with physical disabilities and/or young people.

It seems that the co-occurrence of ADHD and ASD confers additive vulnerability and complexity; hence, the United Kingdom ADHD Partnership (UKAP) convened a consensus meeting, attended by multidisciplinary experts working in the field, to address the complexities of ADHD and ASD from different perspectives, consider the clinical challenges of working with this complex group of patients and how to resolve them. A list of abbreviations can be found at the beginning of the manuscript.

## Methods

Experts in the fields of ADHD and ASD convened in December 2017 in London, UK, for a meeting hosted by the United Kingdom ADHD Partnership (UKAP; www.UKADHD.com). The attendees represented a multidisciplinary group of prescribing and non-prescribing clinical and academic experts with extensive experience working with individuals with ADHD and ASD (covering the fields of psychiatry, paediatrics, general practice, clinical psychology, social work, speech and language therapy, psychotherapy and clinical academic research). We were also fortunate to have representation from the field of education. The meeting included presentations summarising research on the topic. Following the presentations, attendees separated into three sub-groups. Each group was tasked to produce a framework of guidance with specific regards to:

- Identification and assessment of children and adults with both ADHD and ASD
- Interventions and treatments for children with both ADHD and ASD
- Intervention and treatments for adults with both ADHD and ASD

The discussions during the breakout sessions were facilitated by group leaders and summarised by note-takers. The methodological orientation that underpinned the focus of the discussion groups was phenomenological, drawing on the empirical research base and their clinical experience. The sub-group leaders then presented their conclusions to all the meeting attendees for another round of discussion and debate until consensus was agreed.

The entire meeting was audio recorded and later transcribed (sub-group discussions were recorded by note-takers). The medical writer consolidated the meeting transcription, electronic slide presentations and breakout session notes into a draft manuscript. It was then revised by the lead author following further consultation with some authors and other expert colleagues who had been unavailable to attend the consensus meeting. The final draft was then circulated again to all authors to review and to agree the final consensus. The consensus reflects the views of the authors based on their practical experience and published research and provides a unified approach for working with children and adults with co-occurring ADHD and ASD.

## Results

### Identification and assessment

A summary of the consensus reached regarding the identification and assessment of ADHD and ASD when this presents as a comorbid diagnosis is presented in Table 1.

**Table 1:** Practice Recommendations for children and adults with comorbid ADHD and ASD: identification and assessment

1. Due to the level of complexity involved, assessments should only be offered by healthcare practitioners with appropriate training and skills.	
2. A comprehensive assessment should include detailed information about the person’s development and functioning across settings spanning many years (see text for topics). Symptom presentation may change over time for both conditions.	
3. Semi-structured clinical interviews that explicitly map onto diagnostic criteria may be helpful as they guide the assessor to complete a comprehensive developmental and clinical interview.	
4. A helpful precursor to any interview is to ask the parent/carer to look at childhood developmental health records, photographs and school reports and/or to think about key transitions in the child’s life (such as moves from home, change of school).	
5. The assessor should take a parsimonious approach, i.e. they should not ‘double count’ symptoms present in both ADHD and ASD.	
6. It is critical to consider the extent to which the individual’s functioning is age appropriate and obtain examples of how difficulties interfere in their functioning and development at home and in educational or work environments.	
7. Compared with males, females with ADHD may present with fewer disruptive behavioural problems and those with ASD may have lower intellectual abilities.	
8. The assessor should consider the potential impact of cultural issues, e.g. use of eye contact and type of play.	
9. Whenever possible, collateral information should be obtained from independent sources, e.g. parent/carer, teacher interviews, observation in school and/or other settings, adults, perusal of school, college and/or employment reports.	
10. Informants who are family members may have ADHD or ASD (perhaps undiagnosed), which may impact upon their judgement of ‘typical’ behaviour.	
11. Symptoms of both ADHD and ASD may be masked for many reasons, which may cloud clinical judgement, e.g. accommodations may be applied at home and the individual may have developed compensatory strategies, which may minimise deficits in social communication and interaction, and/or skills to ‘camouflage’ difficulties in specific situations or for a brief period of time.	
12. There are high rates of comorbidity associated with both ADHD and ASD, and the assessment should identify whether co-existing comorbid disorders are present. Young children presenting with an initial diagnosis of ASD should be continually monitored through development for ADHD, particularly given that the average age of diagnosis for the two conditions is discrepant.	
13. A risk assessment should be included (see text for topics) together with an assessment of dysfunctional strategies. For example, in young people and adults, these might be alcohol or substance misuse to manage social anxiety or low mood.	
14. Assessment of capacity may be warranted for adults with ASD with severe impairments who require substantial support.	
15. Rating scales are not diagnostic instruments but tools to aid diagnosis and monitor clinical progress. If used to screen, individuals receiving borderline scores (i.e. falling just below cut-offs) should not be excluded from referral for a comprehensive clinical diagnostic assessment.	
16. Norms for many screening tools are often based predominantly on male samples, which may disadvantage their use in females.	
17. Reporting tools such as visual representations of mood states or visual analogue scales will facilitate the assessor to obtain subjective information from individuals who have difficulty identifying or describing their thoughts, feels and sensations.	
18. Observational assessments, including neuropsychological tests, are not diagnostic. They augment clinical decision-making by providing useful information regarding a person’s functioning.	
19. An intellectual assessment should always be considered, together with an assessment of adaptive functioning, and a low threshold applied for their administration.	
20. An intellectual assessment may be helpful for children and young people in school or further education to determine cognitive strengths and weaknesses, establish goals of treatment and target appropriate educational interventions.	
21. For both conditions, uneven cognitive profiles are commonly seen across verbal abilities, performance abilities, working memory and processing speed, reflecting variable strengths and weaknesses.	
22. The outcome of the assessment should include a diagnostic formulation as well as an aetiological formulation that includes protective, predisposing, precipitating and perpetuating factors that inform a comprehensive care plan which takes into account of the needs of the individual and how these may be met across settings (see text for topics). It should also include a Positive Behavioural Support plan, which aims to provide a consistent approach between different caregivers. The care plan should be shared with all relevant parties, as appropriate (including educational establishments, with consent).	

When conducting an assessment for either disorder or when considering the possibility of both co-occurring, it is essential to apply a multifaceted assessment approach that may include the use of standardised and semi-standardised measures. As ADHD and ASD are both complex neurodevelopmental conditions, a comprehensive assessment should include background information (including developmental, clinical and family history, and a current mental state examination), rating scales, a clinical diagnostic interview (including a detailed assessment of symptoms and examples of impairment and, if required, a risk assessment), observational data and an assessment of intellectual ability. Collectively, the information gained from this approach will guide the diagnostic decision. No single point of evaluation or instrument should be conclusive in making a diagnostic decision.

The high rate of co-occurrence between ADHD and ASD means that both conditions should be considered when one of the conditions is present; ASD may confer greater risk for co-existing ADHD where the prevalence of the dual diagnoses appears to be somewhat higher [3, 44, 45]. Indeed, young children presenting with an initial diagnosis of ASD should be continually monitored through development for ADHD, particularly given that the average age of diagnosis for the two conditions is discrepant.

Some individuals with ADHD find their symptoms substantially improve in young adulthood; others continue to sustain symptoms that cause impairment well into middle to late adulthood. In adolescence, people with ASD and/or intellectual disability may experience small improvements in their social functioning, but gains can be limited, and their difficulties and/or impairments persist across the lifespan [26, 51]. Symptoms associated with ‘mild’ ASD may not be recognised (or not prioritised) in children with challenging behaviours associated with severe ADHD; however, this may change with priorities reversing in young adulthood when ADHD symptoms may start to remit for many young people. For this reason, a comprehensive assessment needs to be conducted that obtains detailed information about the person’s functioning spanning many years. It is important to get this right, as a neurodevelopmental diagnosis has the potential to help and guide understanding and necessary support.

It is therefore of concern that assessments (and diagnoses) are made by practitioners with limited training and skills and without a comprehensive appreciation of the variation and idiosyncrasies in the presentation of both conditions. The recent revisions to diagnostic criteria (DSM-5 and ICD-11) have been substantial, and this further emphasises the need for healthcare practitioners to obtain appropriate training for their continuing professional development. Aside from ensuring they access appropriate training, it is essential that practitioners refer to the diagnostic criteria to guide their clinical decision and apply the various clinical tools that are available to support it, rather than rely on a ‘general sense’ of the conditions. In such cases, there is a risk for missed or misdiagnosis. For example, a diagnosis of ASD might not be made because the individual is able to sustain eye contact and will initiate conversations on topics of interest. A diagnosis of ADHD might not be made because the individual does not present with hyperactivity and is able to settle or can ‘hyper-focus’ on a topic of interest or one that provides immediate gratification.

Research on gender differences suggests that girls may be consistently under-identified for both ADHD and ASD [52, 53]. This may be due to multiple reasons. Females with ADHD are reported to have fewer hyperactive/impulsive symptoms and more inattentive symptoms compared with males with ADHD [52, 54]. This may lead to a referral bias as they may not present with behavioural problems that are challenging to manage. Indeed, the lower rates of disruptive behaviour in females with ADHD (compared with males) may contribute to the ‘gender gap’ of 3:1 between males and females respectively [14]. Similarly, there is a ‘gender gap’ in ASD but it is more marked at 4:1, respectively [4]. Females with ASD are more likely than males to have comorbid intellectual disability; hence, those without accompanying intellectual impairments or language delays may not be recognised [4]. It has also been reported that females may acquire superficial learned social skills and exhibit milder restrictive stereotyped behaviour [55], which may mask their underlying social difficulties. The problem is further compounded when rating scales are used as few have separate gender norms, most being predominantly comprised of males. Hence, rating scales may lack the sensitivity and specificity to identify symptoms in females.

Some diagnostic measures have been validated in different cultural contexts [56, 57], but the majority have been developed and standardised in western societies. Cultural issues are likely to impact the assessment, for example, expectations regarding the use of eye contact vary in different parts of the world, and hyperactivity in low- to middle-income countries may be beneficial in some settings but challenging within a post-industrial western educational system [58]. In the criteria for ASD, it seems that ICD-11 will disregard the type of play that children partake in since this may be sensitive to cultural influence but place greater emphasis on the approach they take to play (e.g. whether they follow or impose strict rules), i.e. behaviour which is less culturally specific. Cultural issues may also influence the diagnosis of children with ADHD. A UK study found that children from immigrant Asian families were rated by teachers as much less overactive and inattentive compared with actometer, observation and psychological test variables compared with their white English contemporaries, the rater bias perhaps reflecting higher expectations and/or symptoms not being noticed in Asian children [59].

When conducting neurodevelopmental assessments, it is common for family members to be involved, especially when assessing children, but also for adults where possible. Heritability estimates of both conditions are high (ranging between 70 and 80% for ADHD and 37 and > 90% for ASD) [4, 14], and it is not uncommon for individuals to present for first diagnosis in adulthood prompted by a diagnosis of autism or ADHD in the family. Hence, it is important to be mindful that informants who are family members may also have (undiagnosed) ADHD or ASD and in turn this may impact their judgement of ‘typical’ behaviour. It is therefore important for the assessor to obtain specific examples of behaviour from the informant and use these examples to make clinically informed judgements, rather than relying upon the informants’ perception of what is ‘typical’ or ‘atypical’.

Whenever possible, the assessor should also obtain collateral information from independent sources. A wealth of useful information may be obtained from observing a child in school and speaking directly with teachers. When assessing adults, perusal of school, college and/or employment reports (if available) can be helpful.

Due to the high rates of comorbidity reported to be present in individuals with either ASD or ADHD (see the ‘Background’ section), practitioners should always consider possible comorbid conditions. In general, females are at greater risk of developing internalising conditions (e.g. anxiety, depression) than males, whereas males are at greater risk of developing externalising conditions (e.g. disruptive behaviour disorders). Positive outcomes may be hampered by unrecognised and untreated co-occurring mental health conditions. Intellectual disability is one of the most common comorbidities of both ADHD and ASD, 46% [60] and up to 70% [61] respectively. Adults with ASD and severe impairments requiring substantial support may lack the capacity to make decisions (this is less likely for those with ADHD without co-occurring ASD); hence, an assessment of capacity may be warranted. This needs to be managed carefully and sensitively, especially when there is a discrepancy between the wishes of the individual and those of their family or caregivers.

Symptoms of both ADHD and ASD may be masked for many reasons, which may cloud clinical judgement. Environmental accommodations may be applied at home, e.g. family outings, social events and school trips may be avoided in order to avoid embarrassing emotional ‘meltdowns’ in public. As it is often easier for accommodations to be made at home than at school, this may result in impairments in functioning not appearing to be pervasive across settings. Adults may also make accommodations; adults with ADHD may select occupations that are not ‘office bound’, which maximise the opportunity to be creative and/or stimulated and minimise the need for administration and organisational skills. Adults with ASD may select and be highly valued in occupations that match their special interests and skills.

Symptoms may also be masked by compensatory strategies and coping mechanisms, which may lead to an underestimation of their underlying difficulties. By the time individuals reach adulthood, it is not uncommon for such techniques to have been developed (especially by those with stronger language and intellectual abilities). These usually facilitate the individual to behave in an appropriately acceptable manner in public for a short time and/or in specific settings. These ‘camouflaging behaviours’ usually have an adaptive or functional purpose, for example, adults with ASD may develop a good understanding of social expectations in formal situations (such as a time-limited consultation with medical professionals). Adults with ADHD may employ strategies to remain focused and/or control the urge to fidget during an important appointment or meeting. Nevertheless, it takes great effort to maintain a social façade of this nature for any length of time and doing so may cause the individual to feel fatigued, stressed and distressed.

The strategies and mechanisms applied may not always be adaptive and functional; however, the assessor should be mindful of strategies applied that are dysfunctional. These may be obvious such as drinking alcohol or taking illicit drugs to manage social anxiety or low mood. Others may be less obvious, such as avoiding specific events, settings and/or people, not facing up to difficulties and problems, withdrawal and spending too much time locked away in a room online and/or not engaging in help-seeking behaviours. Some individuals may seek to obtain a social network by forming damaging relationships such as joining a gang, making themselves sexually available, engaging in promiscuous and unsafe sexual practices and/or being used by others for criminal activities.

There may appear to be some symptom overlap across the two conditions, which can complicate clinical assessment. Rating scales may not pick up on qualitative differences in presentation, especially when these are subtle, requiring the assessor to probe for detailed examples of behaviour. For example, when considering abnormal social reciprocity—a person with ADHD may be over-talkative and dominate a conversation, speaking with energy and passion, speak too loud, stand too close, be tangential and frequently change the topic, lose their train of thought mid-sentence, interrupt people’s conversations and illustrate what they are saying with gesture. A person with ASD may be over-talkative and dominate a conversation regarding a special interest, dogmatically stick to that topic and bring the conversation back to it when the other person tries to talk about something else. They may avoid making eye contact, tone and conversation style may seem ‘flat’, and they appear to have limited interest in the other person and lack awareness of social rules and cues (e.g. not asking questions, making inappropriate comments). Both examples indicate limited social reciprocity, but the clinical quality of the deficits is markedly different.

An individual with a co-occurring ADHD and ASD may show a combination of both characteristics in different situations. However, to meet diagnostic criteria for an additional comorbid diagnosis, there must be enough evidence for the second diagnosis over and above what would be expected for the first diagnosis. The assessor therefore should take a parsimonious approach, i.e. they should not ‘double count’ symptoms. For example, if ‘talkativeness’ has been rated as an ADHD symptom, then it should only be re-counted as an ASD symptom if it is distinctly different (i.e. odd and monotonous). Similarly, if invading personal space has already been counted as a symptom of ADHD it should not be counted again as an ASD symptom.

#### Rating scales

Rating scales may be used for two purposes: (1) to screen for a suspected condition and (2) to monitor responses to treatment and interventions. Rating scales are often used to aid clinical assessment, and they are useful tools for monitoring clinical progress; however, when used for screening purposes, it should be borne in mind that they are non-specific markers of potential problems, the results of which should be interpreted cautiously [62]. Rigid adherence to cut-offs is likely to lead to a high proportion of false positives and negatives and unnecessary concerns and anxiety among carer interventionss and caregivers.

There are many rating scales available with varying merits and limitations, and we present in Table 2 some that are in common use in the UK. Some are available in multiple languages and those that are free of charge are indicated with an asterisk (*).

**Table 2:** Supplementary information: rating scales and clinical interviews that are commonly used in the UK (those that are free of charge are indicated with an asterisk)

*Autism-specific rating scales*	
• Social Communication Questionnaire (SCQ) for use with mental age of 2+ [63]	
• Social Responsiveness Scale SRS-2 (age 2½ +) [64]	
• Social and Communication Disorders Checklist (SCDC) (3–19 years) [65]	
*ADHD-specific rating scales*	
• Conners’ Comprehensive Behavior Rating Scales (CBRS) to identify ADHD and comorbid conditions (6–18 years) [66]	
• Conners’ Adult Rating Scales (CAARS) (age 18+) [67]	
• SNAP-IV Rating Scale* (6–18 years) [68]	
• Adult ADHD Self-report Rating Scale (ASRS)* (age 18+) [69]	
• RATE and RATE-C self- and informant-report scales* (for children 8–11 years and adults 16–54 years) [70]	
• The Vanderbilt ADHD Diagnostic Rating Scale (VADRS) [71, 72]	
*Rating scales suitable for both ASD and ADHD*	
• Kiddie-SADS DSM-5 Screen Interview (K-SADS-PL)* (6–18 years) [73, 74]	
• Strengths and Difficulties Questionnaire (SDQ) (2–17 years) [75]	
• The Development and Well-being Assessment (DAWBA) (5–17 years) [76, 77]	
*Autism-specific clinical interviews*	
• Diagnostic Autism Spectrum Interview (DASI)* (age > 2) [78]	
• Autism Diagnostic Interview-Revised (ADI-R) (age > 2) [79]	
• Diagnostic Interview for Social Communication Disorders (DISCO) (age range unspecified) [80]	
• Developmental, Dimensional and Diagnostic Interview [3Di] (highly structured computerised interview; > 2 years) [81]	
*ADHD-specific clinical interviews*	
• ADHD Child Interview (ACE)* and the adult version, ACE+* (5–16 and > 16 years respectively [82, 83]	
• Diagnostic Interview of Adult ADHD (DIVA-2)* (lower age limit not specified) [84]	
*Clinical interviews suitable for both ASD and ADHD*	
• The Development and Well-being Assessment (DAWBA) (5–17 years) [76, 77]	

The Strengths and Difficulties Questionnaire (SDQ) is a widely used brief behavioural screening questionnaire tool that evaluates emotional symptoms, conduct problems, hyperactivity/inattention, peer relationship problems and antisocial behaviour in children age 3–16 years. Translated into over 80 languages, it is therefore a helpful rating scale for use in children with both ADHD and ASD. Depending on the circumstances, there is a charge for using the SDQ.

Specific populations (such as females, younger children higher functioning young people and individuals with intellectual disability) may require adaptations to be made to a rating scale, if one cannot be identified that better addresses their functional abilities. For example, it is common for people with ASD (with or without intellectual disability) to have difficulty identifying or describing their thoughts, feelings and sensations. In such cases, observational tools such as visual representations of mood states, visual analogue scales, sketches and drawings may be useful aids. It is important to be mindful that norms for many screening tools are often based predominantly on male samples, which may disadvantage their use in females (in which case greater emphasis should be placed on carer interventionsal and school reports if female norms are not available).

Rating scales for adult use often rely on self-report. Adults with specific learning or motor difficulties may need support to complete them. Another problem is that some (but not all) individuals may have limited insight into their current and past difficulties (this is the case for individuals of any age, both with and without intellectual impairment). For those with ASD, an additional problem may arise for those with language problems, e.g. they may struggle with seemingly arbitrary phrasing and/or undefined concepts (for example, having to select the frequency of a behaviour from a choice of terms such as ‘occasionally’ and ‘sometimes’). People with ADHD tend to make careless errors due to poor concentration, and it is not uncommon for items to be completely missed out in questionnaires and/or for more than one response to be endorsed on a scale by accident. Hence, questionnaire responses need to be carefully checked and, in some cases, interpreted with caution.

Accepting the limitations of rating scales used for purposes of screening described above when used in clinical settings, there may be merit for using rating scales to screen children in educational settings who are regularly excluded from school and/or who are under-achieving academically. Similarly, it may be helpful to screen children at risk in social care.

#### Clinical interview

The interview should always commence with current concerns as well as background information (including family, past psychiatric and medical history) and move on to obtain a detailed developmental and clinical history that assesses the onset, trajectory, persistence and pervasiveness of symptoms as this will assist with differentiating between ADHD, ASD and other comorbid conditions. A current mental state examination should always be included as this is helpful for indicating the presence of comorbid psychiatric conditions and the assessment of risk. If indicated, some individuals may require referral for development coordination disorder.

It is critical to consider the extent to which the individual’s functioning is both age and developmentally appropriate. A child’s chronological age relates to their date of birth, and it may differ substantially from their developmental age. Their developmental age is the age at which they function emotionally, physically, cognitively and socially. For example, a child may be 12 years of age, but developmentally, they display emotions or behaviours that make them seem much younger. Hence, it is important not to focus solely on a child’s intellectual limitations or chronological age but to be mindful of a child’s developmental limitations more broadly. For example, a child of 12 years may have an IQ that falls within normal limits, but their emotional and social functioning may be highly discrepant with other functioning and developmentally inappropriate. Hence, one has to consider the broader developmental functioning of the child.

Environmental factors may also be salient. It is important to obtain examples of how difficulties interfere in the person’s functioning and development at home and education/work environments. For example, are unhelpful factors and dynamics present, which exacerbate symptoms and affect the person’s ability to cope? Does fluorescent or harsh lighting, hot or cold temperatures, smells of air fresheners or pets, general clutter, or noises from the TV, trains or road traffic have a negative effect? Can these factors be changed or can the individual’s reaction to them be changed?

Semi-structured clinical diagnostic interviews are particularly helpful as they guide the healthcare practitioner to complete a comprehensive developmental and clinical interview. There are fewer clinical interviews to choose from compared with rating scales and, as with rating scales, these have varying merits and limitations. We present in Table 2 some that are in common use in the UK. Some are available in multiple languages and those that are free of charge are indicated with an asterisk (*).

Administering semi-structured and structured interviews can be resource intensive and costly. It can be seen from the above that most clinical interviews specific to autism must be purchased, whereas most interviews specific to assessing ADHD are free of charge. Most require administration by trained interviewers. ADHD clinical interviews such as ACE, ACE+ and DIVA-2 have been developed to directly map onto diagnostic criteria, but most autism interviews have not; hence, if using the ADI-R for example, it is advised that this is done manually (i.e. by cross referencing the information obtained in the interview with the diagnostic criteria). The recently launched Diagnostic Autism Spectrum Interview (DASI) [78] also maps directly onto diagnostic criteria and to our knowledge is the only autism diagnostic tool that is free of charge. Whilst ‘mapping’ the clinical information onto the diagnostic criteria, the persistence and pervasiveness of symptoms should be considered, as well as differential diagnoses. In complex cases, this requires a skilled and experienced multidisciplinary team. ACE and ACE+ are the only clinical interviews that include an integral option for the assessor to apply either DSM or ICD criteria and include a prompt to consider comorbid conditions. For autism, the DASI also includes a prompt to consider comorbid conditions. The DAWBA can be used to assess ASD, ADHD and associated comorbidities at the same time. It is modular in format via a ‘package’ of questionnaires designed to make a range of psychiatric diagnoses that can be completed online (using self- or informant-report, as appropriate) or via interview.

Risk assessment of harm to self and others and from others should be included in the assessment for both ADHD and ASD. The vulnerabilities associated with those who have both conditions may elevate risk further. There is evidence that adolescents with ASD are at higher risk of suicidal thinking, making suicidal plans and engaging in self-harm with suicidal intent than young people without ASD [46]; similar evidence has also been found for individuals with ADHD [84]. Young people with ASD and an IQ that falls within the average range appear to be at particular risk [85]. Although the association is documented to emerge in adolescence, clinicians gave anecdotal accounts at the meeting that this is also a serious problem in younger children. It was a concern of the group that young people with ASD may act on suicidal ideation due to their tendency to rigidly adhere to ideas. Young people with ADHD, by contrast, may become distracted and/or make inadequate plans. They may nevertheless act impulsively on an idea (as may young people with ASD).

A risk assessment should therefore be included for children and young people, as well as adults, that enquires about suicidal ideation; use of illicit drugs/substances and alcohol; antisocial attitudes and behaviours; any harm to self and others, or from others; excessive internet use; unsafe sexual practices; victimisation of bullying and assault; and sexual/financial/social exploitation. For individuals with ASD, a risk assessment should take account of sensory deficits, such as seeming oblivious to pain or to significant temperature changes.

When conducting clinical assessments in adults, a developmental history should be obtained, where possible, which can be difficult when this is self-reported. Whenever possible, this should be provided by an informant, such as a carer interventions/carer, although this may also be challenging due to the passage of time. A helpful prompt may be to ask the carer interventions/carer to look at childhood developmental health records, photographs and school reports and/or to think about key transitions in the child’s life (such as moves from home, change of school). However, the assessor needs to be mindful of the need for symptoms to be consistent and pervasive over time and across settings; difficulties should not be better explained by a situation-specific event that required the child to adapt to substantial change in their life (such as moving home or school). The assessor should also consider whether difficulties are being masked by compensatory strategies and/or ‘accommodations’ (i.e. adjustments) made by others.

In some cases, a carer interventions/carer may not be available, in which case collateral information should be obtained from another close family member. If a suitable informant cannot be identified who knew (and can recall) the individual well during their early childhood, it is valuable to obtain information from an informant who currently knows the individual well (e.g. a partner) as they can supplement self-reported information with an objective perspective. If available, reports from childhood (for example, school, social service and/or previous clinical reports) are likely to be informative.

#### Objective assessments

Objective assessments range from direct observation of an individual in a specific setting (e.g. in clinic, at home or at school) that provides qualitative information about the individual’s behaviour to standardised assessments that compare an individual’s behaviour or performance against norms obtained from a general and/or other specific populations. The assessor should be mindful of caveats that may influence outcomes. For example, if the individual is in a novel environment and/or aware of being observed, he may appear more socially able than usual due to ‘surface skills’ that he is able to apply and manage for a short period of time. In particular, the use of compensatory strategies may minimise deficits in social communication and interaction. With respect to more formal testing, problems with mobility may limit performance and/or the individual may perform relatively well on novel tasks, especially when these are delivered via a mode of interest (e.g. computerised tests). Moreover, a test environment is often arranged to be one that optimises performance (i.e. it is conducted in a quiet room that minimises distraction by an assessor who provides individual attention to ensure the person understands the test instructions and who provides prompts, encouragement and feedback). Hence, outcomes may lack ecological validity as they may not reflect performance in the ‘real world’.

The Autism Diagnostic Observation Schedule Second Edition (ADOS-2) [86, 87] is a widely used semi-structured standardised measure of communication, social interaction, play/imagination and restricted/repetitive behaviours. It is used with individuals who have suspected ASD. The ADOS-2 requires a trained assessor to administer structured and semi-structured tasks and questions to elicit a range of responses in children (including toddlers) and adults. Although the title includes ‘diagnostic’, it is not a diagnostic instrument but a useful measure to be used in support of a clinical assessment. Females may develop superficial learned social skills, which may ‘camouflage’ underlying difficulties or exhibit milder restrictive stereotyped behaviours [55] leading them to score lower on the ADOS-2 compared with males.

An intellectual assessment should always be considered (especially in cases when intellectual impairment or an uneven cognitive profile is suspected). Practitioners should maintain a low threshold for administering the most recent versions of available cognitive tests such as the Wechsler intelligence scales for children or adults (i.e. currently the WAIS-IV and the WISC-5 [88, 89] together with an assessment of adaptive functioning, such as the ABAS-3 [90] or the Vineland-3 [91]). The Kaufman Assessment Battery for Children (KABC-II NU) is a measure of cognitive ability that is culturally fair and includes a non-verbal option and standardisation samples that include people with ASD and ADHD [92]. Cognitive assessments of this nature will inform the psychological treatment approach as those with low intellectual abilities and/or poor verbal skills may respond better to interventions that focus more on a behavioural approach delivered directly to the individual and/or via carer interventionss/carers.

For both conditions, uneven cognitive profiles are commonly seen which may complicate the determination of an individual’s general intellectual abilities (i.e. the Full Scale IQ). In such cases, it is more informative to focus on the four individual intellectual sub-scales or indices that summarise the person’s verbal abilities, performance abilities, working memory and processing speed. The inclusion of an intellectual assessment is particularly useful for both young people in school/further education and adults as it provides information about the individual’s cognitive strengths and weaknesses, which in turn informs goals for treatment by indicating specific areas of cognitive weakness that can then be targeted with appropriate interventions in educational settings.

Neuropsychological testing is neither necessary nor sufficient for a diagnosis of ADHD; however, tests that assess executive dysfunction are helpful in determining deficits in higher order processing skills such as task switching, perseveration, planning, sequencing and organising information. Some have been specifically developed for use with an ADHD population and focus on assessing attention, impulsivity and vigilance in children and adults, e.g. Go-No-Go tasks, Stop tasks and continuous performance tests. Those most commonly used in clinical practice include the Conners’ Continuous Performance Test, third edition (CPT 3 [age 8+]) [93] and the QbTest [94]. These tests are not specific markers of ADHD, but they augment clinical decision-making by providing useful information regarding a person’s cognitive functioning. The assessor should be mindful of the limitations of formal tests and test environments raised above.

#### Outcome of the assessment

The outcome of the assessment should include a diagnostic formulation as well as an aetiological formulation that includes protective, predisposing, precipitating and perpetuating factors that inform a comprehensive care plan which considers of the needs of the individual and how these may be met across settings. This might include suggested pharmacological and/or psychological interventions to manage co-occurring difficulties such as anxiety or low mood, to minimise presenting risks, difficulties in social communication and behaviour and improve cognitive skills, study skills and social skills. For children and young people at school or in further education, a comprehensive formulation of current strengths and difficulties could be shared with educational establishments and other professionals involved in their care, should the young person consent. A comprehensive clinical assessment report should be copied to the patient and referrer. See Table 3 for a summary of suggested content.

**Table 3:** Supplementary information: clinical assessment report content

• Diagnostic and aetiological formulation.	
• Current presenting problems (e.g. physical health (including presence/absence of patient/family contraindicating use of ADHD medication, known genetic anomalies, GI/immune problems, sleep, allergies), comorbid psychiatric conditions and associated problems) and current treatment (psychological/pharmacological).	
• Specific findings from a clinical interview (incorporating information from the individual and informants, if present) applied to either DSM or ICD diagnostic criteria.	
• If administered, findings from observational assessments (qualitative and semi-structured) and/or standardised test results (e.g. rating scales, cognitive and language assessments, neuropsychological tests) that support the clinical decision made.	
• Outcome of risk assessment and consideration of future challenges (e.g. personal, clinical, educational and social transitions).	
• Factors that may lead to the masking or moderation of behaviour in different settings, e.g. compensatory strategies or accommodations at home or school (both functional and dysfunctional), realistic expectations of behaviour and achievement.	
• Consideration of strengths as well as weaknesses (both in terms of personal, family and external networks).	
• Level of support required across settings and how this might be provided or improved.	
• Appropriate interventions (e.g. psychoeducation for the individual, parents/carers, teachers; environmental accommodations that can be made at home, educational and/or other settings; parent-mediated interventions; individual work using a cognitive behavioural paradigm to improve coping strategies and develop skills and determine whether this needs to focus more on behavioural interventions). The appropriate mode of intervention should also be specified, e.g. whether the person can manage individual and/or group interventions or whether these must be applied indirectly via parents/carers and environmental changes (ideally a mixture of both).	
• Further assessment needs, if required (e.g. observation or Functional Behavioural Analysis in specific settings, development coordination disorder)	
• Recommendations for local carer’s assessments for parent/partners, or vulnerable adult assessments for patients, as appropriate.	

### Non-pharmacological interventions for ADHD and ASD

Those with comorbid ADHD and ASD present with additional layers of complexity, often requiring psychological and/or environmental interventions. In order to maximise the likelihood of positive outcomes from any therapeutic intervention, the influence of the individual’s relational and situational context must be considered. This includes both the home and education/work environments. There may be unhelpful factors and dynamics exacerbating symptoms and affecting the person’s ability to cope, such as fluorescent or harsh lighting, temperatures, smells (air fresheners or pets), noises both internal (e.g. TV) or external sources (e.g. trains or road traffic), unclear labels (e.g. for toilets) and/or a cluttered environment with obstacles that they need to navigate. A systemic approach is therefore advised.

Due to the unique presentation and difficulties experienced by children and adults with ADHD and ASD, non-pharmacological interventions must be tailored to meet their individual needs. This may relate to ‘internal’ change that the individual can make themselves (e.g. by developing functional coping mechanisms and adaptive techniques that they can apply) as well as ‘external’ changes that can be made within the environment (e.g. systemic adaptations working with the family/carers and/or school in order to optimise achievement). The approach taken depends on the developmental level of the individual, their clinical and behavioural presentation and their social communication skills.

It is important to ensure that individuals and/or carer interventionss/carers have realistic expectations of the patient’s abilities and what may be achieved by interventions (both medical and non-medical). The rationale for taking medication, if prescribed, needs to be explained (e.g. what it is, how it works, what it does, why they are taking it, what the expectations are). This approach will foster understanding and personal collaboration that will aid compliance.

Due to the high estimates of heritability for neurodevelopmental conditions, some carer interventionss may also have symptoms of ADHD and/or ASD symptoms, so interventions targeted at carer interventionss should be delivered in a way that will facilitate their acquisition of knowledge and skills.

We consider below specific interventions for (1) working with children, young people and their carer interventionss/carers and (2) working with adults. It is important that proposed intervention plans are included in the individual’s care plan and shared with all relevant personnel. Positive Behavioural Support plans focus on providing consistency of interventions between different caregivers or staff and the individual. This will increase awareness and understanding of caregivers and staff of early warning signs (both external as in the example, but also precursory behaviours of the individual that may indicate an emotional outburst is imminent) and develop proactive strategies.

#### Working with children, young people and their carer interventionss/carers

A summary of the consensus reached regarding non-pharmacological clinical interventions for children and adolescents with comorbid ADHD and ASD is presented in Table 4.

**Table 4:** Practice recommendations for children and adolescents with comorbid ADHD and ASD: non-pharmacological clinical interventions

1. Whenever possible, provide psychoeducation with both the young person and their parents/carers taking a lifespan approach. Follow-up sessions are essential to provide information and support and key points of transition (see text for topics).	
2. Ensure that individuals and/or parents/carers have realistic expectations of the child’s abilities and what may be achieved by interventions (both medical and non-medical).	
3. Psychoeducational programmes should differ for children and adolescent populations with the latter including issues relating to transition, sexuality and risk.	
4. Parent/carer support interventions provide a supportive and contained ‘space’ where service-users can meet and share experiences. The focus is predominantly on the parent/carer with the child being the indirect beneficiary (see text for topics).	
5. Acknowledge the difficulties experienced by parents/carers in coping with a child with complex needs on a daily basis by including content that will help the parents/carers to manage their own feelings of isolation, stress, anxiety and depression.	
6. Parent/carer-mediated interventions teach parents/carers to deliver behavioural and environmental interventions to their child. The parent/carer is the agent of change and the child is the direct beneficiary (see text for topics).	
7. A Functional Behavioural Analysis is an observational technique that systematically records the antecedents, behaviours and consequences of behaviour. It is helpful to provide insight into the triggers of challenging behaviour and factors that maintain the behaviour. In turn, this informs the method and goals of treatment.	
8. Cognitive approaches (including cognitive remediation therapy [CRT] and cognitive behavioural therapy [CBT]) are more suitable for young people in adolescence than younger children, although the balance between cognitive and behavioural interventions may need to favour the latter when treating children with ADHD and co-existing ASD (see text for topics).	
9. An increased number of sessions may be required with sessions being delivered at a slower pace, of shorter duration and/or including mid-session breaks. Greater structure and adherence to a clear agenda will help to reduce uncertainty and anxiety. Environmental adaptations may be needed to minimise sensory discomfort and distractions (e.g. sensitivity to light, smells and sounds).	
10. It is helpful to include parents/carers (and teachers if appropriate) to support the young person to apply techniques learned in therapy across different contexts.	
11. In order to avoid disengagement from services, transition planning should occur at least 1 year before a young person moves from child to adult services so that appropriate supports may be identified. A successful transition should involve everyone in the person’s circle of support.	
12. All treatment approaches should be integrated into a comprehensive care plan. This should include a Positive Behavioural Support plan, which aims to provide consistency of interventions (including educational) between different caregivers, staff and service-user(s). The care plan should be shared with all relevant parties, with appropriate consent.	

##### Psychoeducation

Psychoeducation is helpful for improving the experience and long-term outcomes for individuals with ADHD and ASD and their carer interventionss/carers. It may be delivered in various formats: as a ‘stand-alone’ one-on-one intervention delivered to individuals, parents/carers and/or others (such as teachers) or in a group format (most commonly to individuals and/or parents/carers).

In addition to ‘stand-alone’ delivery (either in individual sessions or in group format), psychoeducation should also be included as a precursor to all other interventions provided (both medical and therapeutic). Indeed, psychoeducation is an integral part of all therapeutic interventions and every face-to-face meeting with the individual and/or parents/carers is an opportunity to provide information and education about ADHD and ASD.

The aim of psychoeducation is to provide information about the condition(s) (ADHD and ASD): This might include topics on aetiology, symptom presentation and associated difficulties, common comorbidities, treatments provided or available, and ‘trigger points’ for increased stress (both small scale, such as going on holiday or attending social events, and large scale, such as change of school, moving home, transitioning between junior and senior school, transitioning between child and adult health services, transitioning to further education or work), local resources and support services. When delivered on a one-to-one basis, input from parent/carers should be included in the development of the individual’s care plan in order to ensure that the rationale for the approach taken in the care of the individual is clear and understood by all.

Due to the ongoing need for psychoeducational interventions, a lifespan approach should be adopted; follow-up sessions are essential to provide information, support and key points of transition. Hence, psychoeducational intervention should not be considered to be a ‘one off course’ but ‘drip fed’ over time with reinforcement at regular intervals. However, uptake rates may be low, perhaps because parents/carers perceive psychoeducation as a lower priority than therapeutic interventions. Practitioners at the consensus meeting commented that feedback from parents/carers is that they find the content too generic and/or that they are too busy or too tired to commit to sessions. Therefore, it is important to consider the resources of the family—in terms of time, emotion and support—and structure the content so it is relevant to the needs of the child (and taking account of the child’s chronological age or developmental age). For example, psychoeducational programmes should differ for children and adolescent populations with the latter including issues relating to transition, sexuality and risk.

##### Parent/carer interventions

Most commonly referred to as parenting interventions, there are two types of intervention that may be offered to parents/carers: (1) parent/carer support interventions and (2) parent/carer-mediated interventions. Ideally, an integration of both approaches is likely to lead to better outcomes, especially with the inclusion of a psychoeducational component.

Families may experience enormous strain in having to persistently support a child with co-occurring ADHD and ASD. Parent/carer support interventions are designed to provide a supportive and contained ‘space’ where they can meet and share experiences with others who are in similar circumstances. These programmes may be time limited or ongoing ‘drop in’ meetings. Sessions may introduce some basic behavioural management strategies, but the focus is predominantly on the parent/carer with the child being the indirect beneficiary via the parent /carer feeling better supported and learning to better cope with their own feelings of stress, distress and feelings of isolation. Discussion topics may include improving coping skills (that of the parent/carer and the child), managing stigma, coping with the impact of the child’s behaviour on family relationships, balancing the demands of employment and family life, building routines and structure, introducing visual schedules, building in ‘me time’ and respite care, accessing supportive networks, enhancing competency in advocating for their child, improving safety in the home and community, applying a sensory diet and using rewards sanctions and reinforcers, as well as reminders and prompts.

Parent/carer-mediated interventions are designed to teach parents/carers to deliver interventions to their child. It is therefore technique focused, and the parent/carer is the agent of change with the child being the direct beneficiary. They are usually delivered in a time-limited group format (up to 12 sessions). There is some evidence base for the use of behavioural interventions of this nature in children with ASD, ADHD, oppositional defiant disorder and conduct disorders with findings for improved parenting skills and decreased levels of family distress [95–98].

The aim of these interventions is to support the child to develop skills and reduce maladaptive behaviours. For children with both ADHD and ASD, the content should include greater focus on social communication skills and methods to address disruptive behaviour, aggression, feeding, sleep and toileting problems. The inclusion of environmental adaptations, whenever possible, is likely to maximise success. It is important to acknowledge the difficulties experienced by parents/carers in coping with a child with complex needs by including aspects in the programme content that will help the parents/carers to manage their own feelings of isolation, stress, anxiety and depression. Sessions should be supplemented with written and/or pictorial handouts summarising the information covered and interventions need to be pitched at a realistic level to achieve success. Small gains are better than no gains, and no gains may be the outcome if goals are perceived to be unrealistic or overwhelming.

##### Behavioural and environmental interventions

Following psychoeducational and parenting programmes, and depending on the individual’s needs and presenting difficulties, specific therapeutic interventions may be offered. Depending on the level of ability, these are often behavioural and environmental interventions that are specifically tailored to the child’s needs and modified to account for their unique presentation, comorbid and associated problems. Most commonly those around the child (parents, carers, teachers) are the agents of change in addition to physical environmental modifications being made in order to optimise outcomes.

For both children and adults with ASD and challenging behaviour, environmental interventions are often preceded by a Functional Behavioural Analysis. This is an observational technique that systematically records the Antecedents, Behaviours and Consequences of behaviour (known as an ABC chart). Typically, an individual is observed in a specific setting (or if appropriate across settings) for a period of time and interacting with different people. The antecedents to behaviour are noted in a chart (i.e. what was happening immediately before the behaviour occurred, what makes it worse, what makes it better), and the behaviour itself is described in detail and its consequences (i.e. what happened afterwards, how the individual and those around him/her responded). The recording and analysing of behaviour using this method provide insight into the triggers and motivations of challenging behaviour and factors that may maintain the behaviour. In turn, this informs the method and goals of treatment. For example, in the case of a functional analysis being conducted to identify triggers of severe emotional outbursts, it is determined that these are precipitated by ‘spinning noises’ such as a washing machine or dishwasher. The interventions may include making parents/carers aware of the trigger, removing the child from the environment before the items are turned on, changing the times they are in use (if possible) and/or moving the child to a calming environment to help them reduce feelings of distress in reaction to the noise. Some behavioural interventions have been specifically developed for toddlers with ASD drawing on this approach, probably the most common being the Applied Behaviour Analysis (ABA) [99].

##### Other therapeutic interventions

Individual sessions working directly with the child are sometimes provided, especially for those with severe symptoms and intellectual limitations and/or those who are unable to tolerate group sessions for other reasons (e.g. excessive hyperactivity, poor social communication). These typically draw on ‘traditional’ therapeutic interventions with modifications as appropriate such as the inclusion of visual analogues and materials. They may also be delivered via their teachers and/or parents/carers. Two semi-structured programmes are available to support this type of intervention which have been specifically developed for young children with cognitive, emotional, social and/or behavioural problems: one for individual delivery [100] and the other for group delivery [101–103].

When delivered directly with the child, more sessions may be required but of slower pace and shorter duration and/or including mid-session breaks. Greater structure and adherence to a clear agenda will help to reduce uncertainty and anxiety. The therapist should also consider whether environmental adaptations are needed to minimise sensory discomfort and distractions (e.g. sensitivity to light and sounds). It is advisable to involve parents/carers (and in some cases teachers, if appropriate) since many people with ASD struggle to generalise from one context to another. Parents/carers can support them to apply strategies they have learned in therapy when they are in different contexts.

For those individuals without intellectual limitations, narrative therapy aims to enhance children and young people’s awareness of their strengths and how to utilise them through the sharing and recounting of ‘stories’ the person tells himself/herself about the world. It aims to promote social adaptation, so the person is better able to manage change in their life by strengthening their self-esteem and self-efficacy. This approach has been found to be useful by some services, but its effectiveness has yet to be robustly researched.

An adapted cognitive behavioural therapy (CBT) approach is likely to be more suitable for young people in adolescence than younger children, although the balance between cognitive and behavioural interventions may need to favour the latter when treating children with ADHD and co-existing ASD. Useful topics include social skills training; emotion recognition, ‘reading’ body language and facial expressions; impulse control; emotional control; methods to improve attention and memory; addressing low self-esteem; assertiveness training; coping with peer pressure; identifying and recognising risky behaviours; managing family and social relationships; constructive planning skills; and problem solving techniques.

Adolescence is an important time to support an individual with ADHD and ASD as it is a time they face many transitions, e.g. between health services, between education settings, leaving education, entering the workplace and hormonal and personal body changes. Peer relationships (or difficulties with them) become more salient. Therefore, when working with adolescents, issues relating to sexual development, desires and sexual behaviour should be included, in addition to risk behaviours and attitudes (e.g. antisocial, psychosexual, alcohol and substance use).

Individuals may require support in resolving difficulties that arise within the criminal justice system if their conduct is deemed to reach critical thresholds. The carrying of ‘alert cards’ may help in triggering additional communication supports at moments of crisis. The alert card may specify the disability and provide contact details of family members/carers and/or professionals involved in their care. This simple intervention may be influential in ensuring the individual receives fair and just treatment.

Those people with ASD that do get involved with the criminal justice system, however, may be disadvantaged by some test processes. For example, the probation service in the UK has started using mandatory polygraph (‘lie detector’) testing for high-risk sex offenders under their supervision [104]. The police can also use polygraphs on a ‘voluntary’ basis. The use of the polygraph is controversial due to problems with its validity [105], and this may be amplified for suspects and offenders with autism, who are likely to be particularly vulnerable to erroneous outcomes (both false-positive and false-negative error rates) due to their social communication difficulties.

##### Education and classroom interventions

A summary of the consensus reached regarding educational interventions for children and adolescents with comorbid ADHD and ASD is presented in Table 5.

**Table 5:** Practice recommendations for children and adolescents with comorbid ADHD and ASD: educational interventions

1. School staff should receive specific training to understand how ADHD and ASD impact on the way that children learn in the classroom and interact with their peers.	
2. Specific learning difficulties such as dyslexia, dyscalculia, dysgraphia, and language and communication deficits should be addressed as early as possible with referral to allied health professionals (e.g. speech and language therapists, occupational therapists) so that appropriate interventions can be introduced before they significantly impact on learning, social functioning and/or development.	
3. Anxiety may be the principal barrier to social inclusion and learning. Some children may feel isolated and deeply distressed in school leading to school refusal. When this happens, it is important that educational services develop comprehensive coordinated support plans to help the child and prevent school refusals in advance.	
4. Knowledge and understanding about the social and sensory needs of individual pupils will enable staff to make the necessary adjustments (e.g. avoidance of congested spaces, loud noises) to reduce stress and promote learning and engagement.	
5. Health and well-being topics should be included within the curriculum to avert later risk (e.g. unplanned pregnancy, STDs, substance misuse and mental health difficulties).	
6. School staff may not fully appreciate the ‘achievement gap’ between chronological age and developmental age especially for children in mainstream school education. This needs to be clearly addressed in the child’s Education, Health and Care plan (EHCP), or equivalent, to ensure that curricular demands are appropriate. The EHCP should be developed and regularly updated with input from all those involved in the child’s care, including parents/carers.	
7. Proactive individual planning regarding the transition from primary to secondary education should be made with the student, the school and others involved in the student’s care (if appropriate) in order to minimise the potential negative impact of this event and feelings of stress.	
8. A personalised education plan [PEP] should be developed (see Table 6 for suggested topics) and shared with the healthcare team for inclusion in the individual’s care plan. The care plan should be shared with all relevant parties, with appropriate consent.	

Unfortunately, many children with complex and comorbid presentations may fail multiple times in school before any action is taken to help them. Difficulties may be compounded by late diagnosis or delay in recognition of primary diagnosis, and/or comorbid disorders following an initial diagnosis and lack of awareness of ADHD and ASD. Education professionals in collaboration with health and social care professionals should be mindful of the range of presentations associated with both conditions and sensitive to the less overt behaviours (inattention/daydreaming) which may indicate a level of risk in pupils (especially, but not exclusively, females) who might not be perceived as particularly challenging or oppositional. Screening tools such as the Strengths and Difficulties Questionnaire [75] could be applied in educational establishments to screen ‘at risk’ pupils and guide the type and direction of intervention that may be required (which may be referral for further assessment).

The current evidence base for the effectiveness of school-based interventions for either ASD or ADHD remains poor and is almost non-existent for comorbid presentations. However, the Collaborative Life Skills Program (CLS), a 12-week psychosocial intervention programme for primary school students with ADHD symptoms, has been tested in a randomised controlled trial and shows promise. CLS integrates school, parent and student treatments delivered by school-based mental health providers. Following the program, students from the CLS-assigned schools had significantly greater improvement on parent and teacher ratings of ADHD symptom severity and organisational functioning, teacher-rated academic performance and parent ratings of oppositional defiant disorder symptoms and social/interpersonal skills. During the following school year, CLS-assigned schools had significantly greater improvement on parent, but not teacher, ratings of ADHD symptom severity, organisational functioning and global impairment. Within-group analysis indicated that parent- and teacher-reported post-treatment gains for CLS in ADHD and oppositional defiant disorder symptoms, organisational skills and academic competence were maintained into the next school year [106, 107].

Nevertheless, due to the paucity of research in this area, the guidance below has been largely derived from practitioner observations with the recommendation that benefits are likely to be maximised where support plans are individualised to pupil needs, where comprehensive assessments extend beyond the diagnostic profile and where education staff have the skills and experience to make regular adjustments as each pupil develops (i.e. dynamic assessment of need).

Significant differences exist between primary, secondary school and college settings in terms of both the executive demands made of children and young people, and the capacity of staff to monitor and track pupil progress. In general, the logistical challenges in secondary schools make it difficult to gain an overview of how a student is performing with multiple teachers and across multiple subject areas, and it is thus vital to ensure that all concerned are fully aware of an individual’s needs and understand of the importance of providing regular feedback to the special educational needs coordinator [SENCO], or equivalent. This is particularly important where treatment is being titrated or novel preparations are being trialled, or when there are changes in a pupil’s presentation in terms of their general health and well-being.

It is important that teachers acknowledge that students with ASD and/or ADHD are not labelled as ‘naughty’ or ‘disruptive’ per se as this may set a precedent within the school environment and stigmatise the child. The attitude and approach in managing a student within his/her peer group and the school at large will define the child’s experience in education.

Special attention should be given to periods of transition, particularly from primary to secondary education, and proactive individual planning for this should be thought through with the student, the school and others involved in the students care (if appropriate) in order to minimise the potential negative impact of this event and feelings of stress.

Given that both ADHD and ASD are associated with specific learning difficulties such as dyslexia and dyscalculia, it is sensible to consider how these might impact on learning and to systematically screen this high-risk population as a matter of course. Likewise, any language or communication deficits should be identified early in primary school settings so that appropriate interventions can be introduced before they have a significant impact on learning, social function and development. Allied health professionals (speech and language therapists, occupational therapists) can assess receptive and expressive language capabilities and movement function (e.g. posture, pencil grip) as well as adaptive behaviour functioning and contribute to educational support plans. Children with autism may find generalising functional skills across different contexts particularly difficult. It is therefore important that school staff work in multi-agency teams, especially in cases where the risk of disengagement is high, or pupil needs are complex. Disengagement from school, possibly to the extent of exclusion, rarely benefits the child, and a proactive approach that supports the child both academically and behaviourally may divert the child from a negative course.

Given the known association between language deficits, challenging behaviour and academic under-attainment, staff may benefit from specific training to understand the significance of subtle difficulties in understanding in the way that children both learn in the classroom and interact with their peers. Such difficulties can lead to heightened anxiety and frustration and both academic under-attainment and social isolation (and thus reduced opportunities for social learning).

Similarly, teachers should be aware of any sensory challenges that a child or young person may face. Given that anxiety may be the principal barrier to social inclusion and learning in school settings, understanding both the social and sensory needs of a pupil can enable staff to make the necessary adjustments (e.g. avoidance of congested spaces, loud noises) to reduce stress and promote learning and engagement. Occupational therapists can support schools to provide the sensory diets needed by many children and young people with comorbid disorders.

Pupils with extreme demand avoidant profiles may require a higher level of individualised support in order to sustain their participation in mainstream settings, and school staff should be aware of the high level of neurodevelopmental risk in the pupil population considered to be ‘school refusers’.

It is helpful for teachers to be aware of both the developmental age of any pupil diagnosed with comorbid ADHD and ASD and their cognitive ability to ensure that curricular demands are appropriate. By the early teenage years, pupils with ADHD may function at a level 2 to 3 years behind their peers as a result of delayed cortical maturation and this may help to explain apparent under-attainment, difficulties with task adherence, or problematic behaviours and social isolation [108].

School can be a challenging environment for children with ADHD and ASD (either alone or when co-occurring). At school, children are academically compared with their peers in class (e.g. rated in position of the class, put into streams according to achievement); however, teachers and teaching assistants may not fully appreciate the ‘achievement gap’ between chronological age and developmental age especially for children who are being educated in mainstream school. This needs to be clearly addressed in the child’s Education, Health and Care Plan (EHCP), or equivalent (e.g. a Coordinated Support Plan in Scotland), which in England and Wales is a legal document which describes a child or young person’s special educational needs, the support they need and the outcomes they would like to achieve. The EHCP should be developed and regularly updated with input from all those involved in the child’s care, including parents/carers.

Difficulties may not relate solely to academic performance but also to social demands (managing peer relationships, team sports, free play during breaks in playground, after school clubs and activities). This may be due to a variety of reasons including peer relationship problems, being intimidated and bullied, social anxiety, perceived sense of failure, stigma, difficulties adapting to school routines and/or sensory sensitivities. Children with ADHD and ASD are often perceived as being so ‘different’ from their peers that they become the subject of ridicule and bullying. They may feel isolated and deeply distressed in the school setting, leading to some children refusing to attend school. When this happens, it is important that educational services develop comprehensive coordinated support plans to help the child and prevent school refusals in advance. This may include allowing flexible school attendance or home tutoring, with the aim of gradual reintegration back into school. Prior to that, adjustments may be made in the school setting to support pupils by providing quiet spaces during breaks and lunch times, supported social groups and higher levels of staff supervision during these times. It is important that support staff (e.g. teaching assistants) are both trained in recognising different levels and types of need and encouraged to raise concerns and contribute to planning meetings.

The poor long-term outcomes for this high-risk population suggest that preventative measures may be helpful and necessary in order to avert later risk (such as unplanned pregnancy, sexually transmitted infections, substance misuse and mental health difficulties). The inclusion of health and well-being topics within the curriculum may be beneficial.

For those with co-occurring ADHD and ASD, a personalised education plan (PEP), or equivalent (e.g. Individualised Educational Programme (IEP), Pupil Support Plan, Co-ordinated Support Plan or Child’s Plan in Scotland), is recommended. See Table 6 for a summary of specific educational interventions that may be helpful for children and adolescents.

**Table 6:** Supplementary information: educational interventions for children and adolescents with comorbid ADHD and ASD

*Individualised strategy plan*	
• Assess broader learning needs, including the presence of specific learning difficulties (e.g. dyslexia, dysgraphia, dyscalculia, developmental coordination disorder), sensory processing difficulties and language or communication impairment.	
• Tailor a curriculum which emphasises the development of ‘islands of competence’ in areas of relative strength, e.g. promoting learning through special interests and exploiting talents in areas such as mathematics and information and communication technology (ICT [e.g. coding/programming]).	
• Introduce small group learning and individualised timetables to support learning.	
• Establish academic and pastoral counselling for students.	
• Provide specific guidance on homework and revision strategies.	
*Assistance to aid concentration and reduce anxiety*	
• Establish provision for supervised breaks at certain intervals during examinations instead of extra time to help sustain concentration and reduce disengagement. Although the practice of allotting extra time to complete an assignment or examination has been used with individuals with various psychological disorders, it is not necessarily the best option for those with ADHD and/or ASD.	
• Consider the need for separate accommodation during examinations to reduce sensory overload and exam stress.	
• Use an oral language modifier to explain or re-phrase language in an examination paper and clarify the meaning for the candidate (subject to approval from awarding bodies such as the Joint Council for Qualifications [109] or Scottish Qualifications Authority [110]).	
• Note learning and teaching opportunities that highlight any difficulty a student may have in sustaining attention and retaining information (short-term working memory).	
• Provide immediate feedback and the positive reinforcement of success, given the sensitivity of the reward mechanism in pupils with ADHD.	
• Introduce ‘movement breaks’ or similar to allow pupils an opportunity to engage in physical exercise during the school day.	
• Use of explicit language when teaching or making task demands of students.	
• To reduce anxiety and reinforce expectations, introduce visual timetables that are adapted to the ability level of the student	
*Personal assistance*	
• Plan carefully for the dispensing of medication during school hours to reduce stigma	
• Use assistive technologies (e.g. voice recognition) when handwriting difficulties arise as a result of poor fine motor skills that are often associated with ASD and ADHD.	
• Consider the additional demands being made on some students during field trips, external excursions or any significant changes in routine.	
*Organisational assistance*	
• Provide guidance and support on deadlines and late submissions and regularly check on progress during extended tasks.	
• Provide regular feedback on performance, progress and likely outcomes.	
• Provide support with task prioritisation and time management through the use of task lists, and mind-maps or similar to break down complex topics.	
• Provide support to develop self-checking strategies for coursework and examination submissions.	
• Provide notes, note-making support or access to online resources to support learning for lectures and classes.	
• Provide an executive summary and/or key learning points of lectures and classes.	

#### Working with adults

A summary of the consensus reached regarding non-pharmacological clinical interventions for adults with comorbid ADHD and ASD is presented in Table 7.

**Table 7:** Practice recommendations for adults with comorbid ADHD and ASD: non-pharmacological clinical interventions

1. The general clinical approach for working with adults is similar to that applied to working with children and adolescents, especially for those with intellectual limitations (refer to Table 4).	
2. For those individuals with intellectual disability, the issues of consent and capacity may need to be carefully considered as this impacts on the individual’s decision-making and legal rights. Any information shared with parents/carers must be done with the individual’s knowledge and consent.	
3. It may be necessary to support parents/carers to gain advice from reputable services about guardianship (particularly when intellectual disability has been diagnosed) and the need for long-term financial planning arrangements to be put in place.	
4. Additional support and guidance may be required for individuals with ADHD and ASD who become parents.	
5. Psychoeducational interventions should be provided to both the individual and carers.	
6. Individuals may benefit from attending peer group support interventions (i.e. a peer version of the parent/carer support interventions described in the child section).	
7. Cognitive approaches (including cognitive remediation therapy [CRT] and cognitive behavioural therapy [CBT]) are likely to be effective interventions for adults with ADHD and ASD (see text for topics). Specific adaptions may be needed for individuals with social communication and intellectual limitations, including greater support and supervision by the therapist.	
8. Adults may be better able to cope with group delivered treatments than children and adolescents, especially when specific adaptations are arranged, for example by increasing the number of group facilitators to ensure individual support can be provided.	
9. Support strategies and planning are needed for transition between child and adult services. In addition, salient life-path transitions (e.g. parental bereavement) should trigger enhanced planning, as should geographical re-location or major relationship changes.	
10. As for children, interventions should be integrated into a bespoke collaborative care plan for the individual, including a Positive Behavioural Support plan to ensure consistency of interventions (including educational) between different caregivers, staff and service-user(s). The care plan should be shared with all relevant parties, with appropriate consent.	

Parents/carers are recognised as a valuable source of expertise in the care needs and specific educational and occupational challenges faced by their child with co-occurring ASD and ADHD. However, parents/carers may experience a sense of exclusion when their child is transitioning into adulthood. This is particularly true if the individual wishes to advocate more for themselves and restrict access to information and decisions regarding their care. Services need to manage the interface between an individual’s right to confidentiality and the need to draw on the expertise that parents/carers have developed over time. Any information shared with parents/carers must be done so with the individual’s knowledge and consent. The issue of guardianship may need to be considered and long-term financial planning arrangements put in place (e.g. setting up a trust fund), particularly when intellectual disability has also been diagnosed. In such cases, signposting to reputable services that can provide advice on these topics may be helpful.

Poor timekeeping and organisation skills may lead to disengagement from important services (e.g. GP, mental/physical health, dentist) and additional structure may be necessary to sustain appointments, treatment regimens and medication (e.g. use of text, phone app reminders). There may often be difficulties in self-regulating behaviour and decision-making, leading to poor lifestyle choices, which may result in poor physical and mental health (e.g. anxiety, depression, forgetting to take medication, dental care), poor financial management, homelessness and social isolation.

Many individuals with ADHD and ASD become parents themselves. However, for a minority, their capacity to support children may be questioned if appropriate support and guidance is not provided. Their commitment to family welfare may be misconstrued if, for example, problems with planning and organisation are interpreted as evidence of neglect. If unnecessary disruption to family life is to be avoided, all professionals involved need to understand how ADHD and ASD may impact on the person’s daily functioning in order to identify the most appropriate types of support that may be required.

There is a dearth of research on the needs of adults with ADHD and ASD who are negotiating transitions in later life, e.g. in middle-age, at retirement and in geriatric patients. Professionals in primary care settings should be conscious of the current under-diagnosis of both conditions in the adult population and mindful of the risks that may become evident in clinical settings, as well as the treatment options available. Likewise, social care professionals may benefit from understanding the range of presentations that these cohorts may demonstrate when demands are made for their services.

The approach for working with adults is similar to that applied to working with children and adolescents, especially for those with severe intellectual impairments (i.e. by providing systemic support and psychoeducation, functional behavioural analysis and the implementation of behavioural environmental interventions, and the provision of individual cognitive behavioural interventions, when appropriate). There are however some key adaptations that will be addressed here. As for children, interventions should be integrated into a bespoke collaborative care plan for the individual.

##### Psychoeducation

The management of consent and capacity may need to be carefully considered in adults as it impacts on the individual’s decision-making capacity and legal rights. It also needs to be managed sensitively, especially in the presence of conflicting perspectives and opinions. Even in the absence of issues of consent and capacity, there may be discrepancies between how the individuals themselves want to live and the wishes of their family or carers. In such cases, there needs to be close communication with families and carers, often involving psychoeducation with both the individual and carers. Indeed, there may be a need to work systemically on family relationships and/or with carers on placement rather than applying a solution-focused approach, e.g. with a focus on values and trust, and establish who to go to for what information and guidance.

Positive Behavioural Support planning is strongly recommended to provide consistency in how carers and staff work with the individual. This will enable them to better understand subtle differences in presentation, identify early warning signals of specific behaviours and develop proactive strategies of prevention and/or intervention.

For adolescents and adults, it is important that individuals develop common sense practices regarding unsupervised use of medications (if appropriate). This may be achieved by providing information about how the medication may impact on their performance in adult activities (including work) and the risks of not taking the medication. They should also learn about the consequences of sharing or selling medications to others, both from a legal perspective as well as from a health perspective (i.e. the potential harm that may arise from supplying medications to others).

Psychoeducation for young people and adults should also address the use of alcohol and illicit drugs, impulsivity, antisocial attitudes and behaviours and their potential outcomes. Sex education is a topic that is sometimes excluded, yet these young people are experiencing sexual feelings and desires. They may engage in promiscuous behaviour to gain friends and, due to their vulnerability, they may be at risk of sexual exploitation. Often, these are issues that they have never spoken about and it may be necessary to ‘give them the language’ to articulate their thoughts and feelings, supplemented with visual analogues and materials and in some cases with anatomically correct dolls.

Pregnancy rates for young women with ADHD are elevated [111, 112] suggesting that this may also be a risk for females with both ADHD and ASD. As young parents themselves, they may need a great deal of support else they may become increasingly vulnerable, particularly if they are the sole caregivers for their children. Hence, specific tailored assessments, support plans and social interventions are needed for this vulnerable group. There is currently limited literature on this topic and more research is required.

##### Psychological interventions

Group work may be challenging for some people with ADHD and ASD, especially those with intellectual limitations, as they may lack concentration and struggle with the social communication demands of group work. They often find it difficult to settle and inhibit their urge to interrupt. Nevertheless, adults may be better able to cope with group delivered treatments than children and adolescents, especially when specific adaptations are arranged, for example by increasing the number of group facilitators to ensure individual support can be provided during group meetings. If possible, it is preferable for individuals to be grouped in some relational way, e.g. by age and/or gender. For those who are unable to tolerate a group format, some content may be appropriately adapted and provided on an individual basis.

The R&R2 for ADHD youths and adults programme, which has a good evidence base when delivered in group sessions in the community [113–115], has been successfully adapted in adults with moderate intellectual impairment. The programme covers topics of relevance to both ADHD and ASD, including the development of prosocial competence skills, cognitive and executive functioning skills, critical analysis and consequential thinking, emotional control, negotiation skills, social problems solving skills, assertiveness training and moral reasoning skills.

The R&R2 is tailored to the needs of its recipients, and compared to the original R&R, it includes more visual material, simpler language and some modified examples and exercises [116].

In addition to the structured or semi-structured group interventions described above, individuals may benefit from attending peer group support interventions (i.e. a peer version of the parent/carer support interventions described in the previous section).

There is evidence that interventions adopting a cognitive paradigm are effective interventions for adults with ADHD (e.g. those drawing on cognitive remediation strategies to address core symptoms and those drawing on CBT strategies to address associated mental health and social problems), and there is increasing evidence for its use with adults with ASD [117–122]. Although there is no published evidence base yet for modified cognitive interventions for adults with both ASD and ADHD, the consensus was to endorse this treatment paradigm in individual sessions for adults with both conditions, provided the therapist has expertise in working with adults with ASD and ADHD. The content of therapy needs to reflect topical issues of importance (to be determined by the patient and the therapist together), such as symptoms of ASD/ADHD, anxiety, low mood, friendships, primary-to-secondary school transitions, sexuality, puberty, management of intimate relationships, substance and alcohol misuse, pregnancy and parenting.

As when working with children, there may need to be specific adaptions to meet individual needs associated with varying social communication and intellectual abilities, including greater support and supervision by the therapist. The content of modified CBT sessions may need to be more practically and behaviourally oriented and include topics such as goal setting, sequencing information and priority setting, planning skills, time management and organisation, budgeting and managing finances, social relationships and social communication skills, problem solving skills, understanding and recognising body language, emotional dysregulation and impulsive behaviour, methods to improve concentration and memory skills. Specific strategies may also need to be taught for low mood, anxiety management, frustration and anger. As when working with young people, in some cases, it may be helpful to involve a family member or carer in the treatment to support them to apply strategies they learn in therapy to different contexts (as people with ASD may struggle to generalise from one context to another). If an adult patient does not specifically request this level of support, their consent needs to be obtained.

##### Further education, career advice and occupational skills training

A summary of the consensus reached regarding educational and occupational interventions for adults with comorbid ADHD and ASD is presented in Table 8.

**Table 8:** Practice recommendations for adults with comorbid ADHD and ASD: educational and occupational interventions

1. Career officers, special educational needs coordinators and occupational therapists should collaborate to ensure young people are directed appropriately towards realistic career goals and independent living.	
2. Educational support services should be aware of potential challenges for students in further education, e.g. elevated levels of anxiety, difficulties in living independently, sleep disruption, social isolation and substance use (see also Table 5).	
3. Standardised disability provisions in tertiary education settings (e.g. the practice of allotting extra time to complete an examination) may not be appropriate for individuals in this group as this extends the total examination time. They may be better supported by receiving a (supervised) mid-point break.	
4. Individuals will require support with applications and interviews, and in negotiating the recruitment process.	
5. Voluntary and supported work placements will help individuals to gain an understanding about the expectations of being in a work setting.	
6. Educational establishments and employers should ensure that staff receive training in neurodiversity to improve general understanding among colleagues.	
7. In the UK, there is legislation that requires reasonable adjustments to be made at work for individuals with a disability in order to avoid the potential for disability discrimination.	
8. Both educational establishments and employers should make expectations explicit and communicate clear goals, sub-goals and deadlines in order to avoid confusion.	
9. Coaching and support may be required to help young people manage their personal finances and plan for their financial commitments.	
9. As for children, where appropriate, a personalised education plan [PEP] should be developed (see Table 9 for suggested topics) and shared with the healthcare team for inclusion in the individual’s care plan. The care plan should be shared with all relevant parties, with appropriate consent.	

The educational approach for young people in further education is not dissimilar to that outlined in the previous section focused on the education of children. Across the contexts of both further education and occupation, each area of disability may magnify the other and multiply the number of potential educational and occupational challenges for a person with both ADHD and ASD. Other additional comorbidities and specific learning difficulties (e.g. dyslexia, dyspraxia, dyscalculia) are also likely to contribute to poorer outcomes.

Transition planning should occur at least 1 year before a young person moves from adolescent to adult services so that appropriate supports may be identified, and a range of potential positive destinations recognised [123, 124]. It is important to consider the strategies and person-centered planning services that can be applied to support transitions (between services, between education settings, leaving education, entering the workplace, between employments, independent/semi-independent living etc.). A successful transition should involve everyone in the person’s circle of support. Significant life-path transitions (e.g. parental bereavement) should also trigger enhanced planning, as should geographical re-location or major relationship changes.

All young people and adults require career advice, and for this vulnerable group, who may have limitations but also strengths in what they can achieve, it is essential that career officers, special educational needs coordinators and occupational therapists collaborate to ensure the young people in their care are directed appropriately towards aspirational and realistic career goals and independent living.

Standardised disability provisions in tertiary education settings (e.g. the practice of allotting extra time to complete an examination) may not be appropriate for individuals in this group. Student support services should be aware of the difficulties that might arise with elevated levels of anxiety, difficulties in living independently, sleep disruption, social isolation and substance use. The groups’ recommendation is to consider a holistic assessment of needs, informed by, and incorporated into a personalised education plan or equivalent (see also section on this topic in the child and adolescent section). See Table 9 for a summary of specific educational and occupational interventions that may be helpful for adults.

**Table 9:** Supplementary information: educational and occupational interventions for adults with comorbid ADHD and ASD

*Individualised strategy plan*	
• Guidance on course/occupational choices to determine the ‘best fit’ with the student’s aptitude and personal needs.	
• Ensure proactive pastoral support is available from professionals with the skills and experience to facilitate communication.	
• Provide opportunities for the student to learn about their own difficulties and strengths to facilitate the adoption of self-help strategies and reduce dependence on others or the risks of disengagement.	
• Ensure the student knows how to gain additional support if difficulties arise with accommodation arrangements.	
• Consideration of the supports necessary where cooperative/collaborative tasks may be set.	
• Liaison between services where students have complex needs arising from health problems (e.g. adherence to treatment programmes, substance use, mental health difficulties).	
*Assistance to aid concentration and reduce anxiety*	
• Allow supervised breaks at certain intervals during examinations instead of extra time (see also advice in child and adolescent section).	
• Environmental modifications to reduce sensory demands or to meet sensory needs (e.g. in terms of lighting and noise).	
• Support during unstructured periods of the day and access to quiet areas in order to reduce social anxiety.	
*Personal assistance*	
• Use of an oral language modifier to explain or re-phrase language in an examination paper and clarify the meaning for the candidate (this is used by the Joint Council for Qualifications [109]).	
• Recognition of the additional demand and struggle for students when making field trips, external excursions and/or activities that involve any significant changes in routine.	
• Training in and access to assistive technologies (e.g. voice recognition).	
• Access to support if difficulties arise with performance and/or interpersonal relationships.	
*Organisational assistance*	
• Explicit instructions and guidance provided in a visualised format (assignment plans, timetables, deadlines, changes in routine).	
• Provide regular proactive follow-up and support to ensure students are coping academically, socially and emotionally.	
• Arrange work-experience opportunities with a recommendation for mentoring and/or a peer ‘buddy support’ system that provides supportive monitoring and guidance.	

Positive and creative approaches should be used to help individuals to spend their time effectively and identify job opportunities. This may include voluntary and supported work placements in order for the individual to gain an understanding about the expectations of being in a work setting. For those able to tolerate them, shared skill groups may help in the training process as well as provide a setting to develop social communication skills and reduce isolation. Formal professional support in this respect is not routinely provided, however, and it often falls to parents/carers or friends to provide guidance and support in navigating occupational expectations.

Those with co-occurring ADHD and ASD may often require support with applications and interviews, and in negotiating the recruitment process. This will also involve consideration of the best way and time to disclose the person has a disability. Occupational testing may include objective tests that require a person to consider interpersonal scenarios and these may not be appropriate for use with people with ASD. One such assessment is the Civil Service Judgement Test, which is commonly used in the UK for the recruitment of staff by the civil service [125]. People with ASD may be disadvantaged by both the content and process of this type of assessment, which includes a requirement they consider online interpersonal scenarios and rate how they would respond (from a 4-point multiple-choice format). This requires the applicant to infer information such as the beliefs, intents, desires, emotions and/or knowledge of those interacting in these brief scenarios (presented in text only or by both text and video). This process, known as ‘theory of mind’, may be challenging for some people with ASD due to their difficulty attributing mental states to both themselves and to others. A person with ASD may be hampered in their ability to decide on the best course of action because they have insufficient information to consider and select a response from fixed options (which may be perceived as arbitrary undefined concepts, such as ‘counterproductive, ineffective, fairly effective, effective'). In ‘real life’, they would be able to draw on environmental cues and contextual information.

Many young people and adults struggle with daily demands which challenge their executive functioning leading to difficulties with punctuality, time management, organising and prioritising work, managing their workload and meeting deadlines. Helpful supports at work include mentoring, pairing with a more experienced ‘buddy’ peer, supportive monitoring and adopting technological aids such as apps and generic software applications. To avoid confusion and/or misguided task prioritisation, it is recommended that expectations and information is explicitly communicated to the employee (both verbally and in writing).

Many occupations involve an interpersonal aspect (e.g. dealing with the public, team working with colleagues, attending meetings, giving presentations, appreciation of and adherence to line-management systems). Individuals with ADHD and ASD may struggle to manage workplace relationships, especially those who actively avoid social communication; lack an appreciation of ‘office politics’, occupational expectations and boundaries (which are often not explicit); and/or do not fully understand the need to adhere to established hierarchies. They may become very anxious, irritable and in some cases aroused and confrontational. They may need help to resolve differences with colleagues before they escalate to more formal disputes. Workplace disputes (both performance-related and interpersonal-related) may be avoided by coaching to understand (1) the organisational structure of the work setting and (2) the expectations of how to dress and behave at work and how this differs from when at home or when socialising with friends. These problems are exacerbated when ADHD and ASD co-occur, resulting in more complex needs and service provision.

Workforce tolerance may be enhanced by providing training in neurodiversity to improve general understanding among colleagues. Employers need to be mindful of equalities legislation, such as the UK Equality Act, 2010, Autism Act 2009 (the first ever disability-specific legislation in England) and Think Autism 2014, and the importance of making reasonable adjustments in order to avoid the potential for disability discrimination.

Some individuals with ADHD and ASD will enjoy a level of independence in terms of their occupation and living arrangements, but it is not uncommon for them to struggle with managing their personal finances. Coaching support may be necessary to help them to learn how to plan and budget for financial commitments to ensure that essential bills (e.g. rent, utilities, phone) are paid on time and/or, if necessary, reduce and amalgamate debts.

### Pharmacological treatment for ADHD and ASD in children and adults

A summary of the consensus reached regarding pharmacological interventions for ADHD and ASD when these conditions present as a comorbid diagnosis (in both children and adults) is presented in Table 10.

The pharmacological treatment for co-occurring ASD and ADHD in children and adults is no different from the treatment of each disorder separately. In the UK, the National Institute for Health and Care Excellence (NICE) guidelines should be followed for the pharmacological treatment of ADHD as a separate condition; medication is commonly offered to treat ADHD symptoms (usually stimulant and non-stimulant medications) in both people with ADHD and those with co-occurring ADHD and ASD. ADHD medications should not be offered to treat people with ASD (without co-occurring ADHD) as there is no evidence for a positive effect in this population [126–128].

**Table 10:** Practice recommendations for children and adults with comorbid ADHD and ASD: pharmacological interventions

1. In children, pharmacological intervention should be preceded by behavioural observation and psychological intervention as first-line treatment.	
2. If psychological/environmental interventions fail in children, then ADHD medication may be helpful for treating symptoms of inattention, hyperactivity, impulsivity, aggression, irritability and emotional lability.	
3. Adults are often prescribed medication prior to psychological interventions, as these may be less commonly available.	
4. Official guidelines (e.g. NICE) should be followed for the pharmacological treatment of ADHD and ASD as separate conditions. There are no recommended pharmacological treatments for core symptoms of ASD per se.	
5. Second-generation antipsychotics may be used with caution for the short-term treatment of irritability in children and adolescents with ASD, although behavioural and environmental interventions are first-line treatments.	
6. Given the high rates of comorbidity associated with both ADHD and ASD, medication should be considered to treat comorbid conditions such as anxiety, mood problems and sleep disturbance.	
7. Close attention should be paid to side effects of any pharmacological treatment due to the increased likelihood of their occurrence in this group.	
8. A ‘low and slow’ approach to titration should be adopted as people with both conditions may be more treatment resistant and more sensitive to the effects of medication (especially with respect to side effects). Medication should be given for the shortest time possible and monitored carefully for the duration of treatment.	
9. Good lines of communication should be established between school and child psychiatric/paediatric services (as appropriate) for purposes of monitoring treatment effect in educational settings.	
10. Due to sensory issues and physical difficulties, individuals with ASD may be unable to consume medications in tablet form (e.g. difficulty swallowing, aversion to tablets). In this case, liquid preparations should be prescribed.	
11. A cautious approach should be taken when prescribing stimulant medications to older adults or other individuals with a history of cardiac problems.	
12. Pharmacological treatment is not advised for women with ADHD and ASD during pregnancy.	
13. With individuals for whom informed consent may be an issue, the use of adapted behavioural observation and reporting tools such as visual representations or visual analogue scales may be helpful.	
14. Clear goals and outcome measures should be specified and monitored to determine the effectiveness of medications prescribed. The reporting tools described above may also be helpful for individuals who have difficulty identifying or describing their thoughts, feelings and sensations in order to facilitate subjective reports of treatment outcome and side effects.	
15. All treatment information should be documented in a medication management plan for integration with a bespoke collaborative care plan for the individual, including a Positive Behavioural Support plan to ensure consistency of interventions (including educational) between different caregivers, staff and service-user(s). The care plan should be shared with all relevant parties, with appropriate consent.	

Indeed, there is no orthodox pharmacological treatment for ASD in either children or adults that has an evidence base for treatment effect [128]. Antipsychotic medication for core ASD symptoms has been used widely in certain countries; however, the evidence base supporting its use in this population is negligible and the risk of adverse effects is high. The second-generation antipsychotics, particularly risperidone and aripiprazole, have been used for the short-term treatment of angry outbursts in children and adolescents with ASD, some of whom will have ADHD [129]. Low doses, regular reviews and short-term use are advised. However, there are variable degrees of official approval for their use between countries and behavioural and environmental interventions are first-line treatments. Selective serotonin reuptake inhibitor [SSRI] medications are not clearly helpful for treating repetitive behaviours in children and adults with comorbid ASD and ADHD [130].

When an individual has a diagnosis of both ADHD and ASD, the trigger point for prescribing medication is when the ADHD symptom presentation is severe and/or associated with impairments that hamper personal, social and educational development and achievement. However, given the substantial symptom overlap between the two conditions, when an individual (child or adult) presents with both, there is merit in commencing with psychological/environmental interventions to treat the associated difficulties of ASD, monitor outcomes and review the need for medication as appropriate. In childhood and adolescence, should psychological/environmental interventions fail, then ADHD medications (stimulant and non-stimulant) are helpful for the characteristic symptoms of inattention, hyperactivity and impulsivity when these are clearly evident (e.g. distinguishing agitation for hyperactivity) and arise from ADHD [131–133]. However, in adults with co-occurring ASD and ADHD, medication to treat their ADHD should be offered as appropriate, with regular monitoring, and should not wait until psychological treatment has been completed. This is especially the case where there is a shortage of specialised ASD/ADHD adult psychology services and where medication for ADHD may be a more immediately available treatment option. Medication for ADHD may also enable adults to better concentrate and maximise benefits from psychological treatment.

Prescribers need to be mindful of the presence of conditions or problems co-existing with combined ADHD and ASD and, if appropriate, prescribe medication, e.g. melatonin may help with the insomnia associated with stimulants. An SSRI may be prescribed to treat depression and/or curb anxiety, though should be used with caution, e.g. SSRI's can interact adversely with amfetamine and in the USA their dispensed packaging carries a 'black box' warning about suicidality in the young, (although this practice is not paralleled in te UK or Europe). The use of second-generation antipsychotics to reduce aggressive behaviour, especially in association with comorbid oppositional defiant or conduct disorder, is mentioned above. There is only limited evidence to suggest that stimulants worsen tics [134], although the possibility of an increase in tics in some individuals should not be ignored and medication adjusted accordingly.

Treatment with (any) medication should not be given to compensate for deficits in the individual’s environment, such as inadequate staffing levels or lack of training and skills. Medications should only be used for the shortest time possible with clearly specified goals and measures of effectiveness in place. The outcomes of treatment should be closely monitored, including vigilant monitoring for side effects. Any medication that is not effective should be stopped.

A significant proportion of people with ASD may have difficulty identifying or describing their thoughts, feelings and sensations, which may cause difficulty with obtaining subjective reports of treatment outcome and side effects (even by those without intellectual disability). This may be facilitated by visual representations of mood states, visual analogue scales, sketches and drawings. These methods may also assist practitioners to obtain informed consent in cases when this is problematic.

When treating people with co-occurring ADHD and ASD, practitioners need to be particularly mindful of the following:

- There is limited data on treating patients who have both ADHD and ASD with ADHD medications, and a careful and circumspect approach should be taken when treating children and adults with co-occurring ADHD and ASD (in comparison to prescribing for ADHD alone). Prescribers should ‘start low and go slow’ as people with the both conditions may be more treatment resistant and more sensitive to the effects of medication, especially with respect to side effects. Some individuals may have lower thresholds for experiencing side effects from medication, which include sleep difficulties, decreased appetite, tics, mood volatility and irritability [131].
- In collaboration with the child and parents/carers, clear goals and outcome measures should be specified and monitored to determine the effectiveness of medications prescribed, including medication for aggression, anxiety and sleep problems.
- Due to sensory issues and physical difficulties, individuals with ASD may experience difficulties with consumption of the medications in tablet form (e.g. difficulty swallowing, aversion to tablets). Many medications are available in liquid or sprinkle preparations.
- Young people with ADHD are at higher risk of teenage pregnancy [111, 112], and it is not advisable to treat pregnant females with stimulant medications. It is important that alternative support is set in place for those who are taken off medication for this reason (see non-pharmacological interventions section).
- A medication management plan should be provided to the individual and/or parents/carers, as appropriate, detailing information about the individual’s pharmacological treatments. This involves specifying the indication for the use of medication, the medication used, measures of effects and side effects, and the plan for stepwise adjustments of dosage and drug switching or augmentation, should that be necessary.

## Conclusions

The co-occurrence of ADHD and ASD is common, and the presence of both conditions exacerbates difficulties above those experienced by either condition alone. The identification and assessment of ADHD and ASD is complex due to shared aetiologies, overlapping symptoms and shared characteristics. The intervention and management of both conditions is complicated by the core features of both disorders and by co-occurring neurodevelopmental and mental health conditions. These may have a significant impact on engagement, effectiveness and outcomes of both pharmacological and psychological interventions. Neurodevelopmental conditions are often missed in children and adults, especially when they present with high rates of comorbidity that healthcare practitioners are more readily able to recognise, e.g. anxiety and depression. This is further complicated by the change in symptom presentation as young people grow up, with reported decline in hyperactive/impulsive symptoms in ADHD and improved social communication in ASD. These ‘improvements’ however may be offset by the development of additional mental health problems and associated impairments that persist into adulthood.

Hence, UKAP hosted a meeting of experts in ADHD and ASD from a range of disciplines with the aim of reaching a consensus regarding the most appropriate methods of identifying and assessing co-occurring ADHD and ASD. We further sought to agree the most effective interventions and treatments for ADHD and ASD in children and adults. The consensus recommendations for practice are summarised in Tables 1, 4, 5, 7, 8 and 10.

Barriers to achieving positive outcomes exist within the clinical, social, and educational spheres. These include poor access to care; lack of training for healthcare staff, teachers and allied professionals; inadequate knowledge about female presentation; lack of care coordination and poor inter-agency working; high rates of comorbidity and functional problems that limit the individual’s ability to engage effectively with a service; inability of the individual to advocate for themselves; stigma (even within the family); need for age-appropriate psychoeducational initiatives; poor coping strategies and substance misuse issues which may significantly impact on an individual’s ability to engage with a service and/or in specific interventions. Unfortunately, some barriers are a result of rigid adherence to the same clinical plan that has been in effect over many years without modifications being made in line with the developments in presentation and needs of the child or adult. A willingness to make changes to accommodate the varied needs of individuals with co-occurring ADHD and ASD over the years will help to eradicate the barriers.

In view of their complex needs, children and adults with co-occurring ADHD and ASD are ‘resource intense patients’ and the experience for the service-user is often that they are passed from service to service. This delays the individual and parents/carers being able to access appropriate treatment and support, sometimes for many years. Individuals with ADHD and ASD should not be denied proper assessment, especially when a screen indicates this is warranted, in order to ensure that their fundamental needs are identified and met as appropriate. Services are better geared to support individuals with intellectual disabilities (i.e. with specialist inpatient services or residential community care settings), but community outpatient services for those without intellectual disabilities are fewer, especially for adults, leaving service-user led charity and support services to ‘fill the gap’. Given the amount of ‘common ground’ shared by those with ADHD and ASD, the implementation of integrated neurodevelopmental services with comprehensive input from a multidisciplinary team working across community and residential settings will be an efficient, cost-effective and user-friendly provision of service and lead to better outcomes for all concerned. This model has been successfully implemented by the Integrated Children’s Service (ICS) in parts of the UK. More and better service provisions are urgently required to meet the complex needs of those with co-occurring ADHD and ASD.

There is a clear need for training about ADHD and ASD, both individually and as comorbid conditions, in health care and allied services. Unfortunately, despite international clinical guidelines to the contrary, ‘lay’ scepticism and resistance to the validity of ADHD may affect perceptions of care needs. Due to the complexity of the presentation when both conditions are present, inter-agency collaborations are required to ensure a successful provision of care drawing on the expertise of professionals working in social care, health and education services. When appropriate, multi-agency input should be included in the individual’s care plan and shared accordingly. Aside from training in the recognition of ADHD, ASD or both conditions in children and adults, training needs to be provided about the additional comorbidities that may be present, gender differences in presentation, appropriate assessment protocols and the referral process, the treatment and interventions available (both medical and psychological), the needs of family and caregivers, inter-agency collaborations and key transitional points of need across the lifespan.

It is hoped that this consensus will support healthcare and allied professionals across a range of disciplines to effectively identify and treat individuals with ADHD and ASD. Accurate and timely diagnoses and appropriate interventions will undoubtedly increase positive outcomes across the lifespan. There is a dearth of research on ADHD and ASD as co-occurring conditions, including gender differences and long-term outcomes (both with and without treatment). In particular, further research is needed to establish an evidence base for the most effective treatments for children and adults, both males and females, who have both conditions.

## Abbreviations

3Di: Developmental, Dimensional and Diagnostic Interview
ABC: Antecedents, Behaviours and Consequences (chart)
ACE: ADHD Child Evaluation
ADHD: Attention deficit/hyperactivity disorder
ADI-R: Autism Diagnostic Interview-Revised
ADOS-2: Autism Diagnostic Observation Schedule, second edition
ASD: Autism spectrum disorder
ASRS: Adult ADHD Self-report Rating Scale
CAARS: Conners’ Adult Rating Scales
CBT: Cognitive Behavioural Therapy
CLS: Collaborative Life Skills Program
CNVs: Copy number variants
CBRS: Conners’ Comprehensive Behavior Rating Scales
CPT 3: Conners’ Continuous Performance Test, third edition
DASI: Diagnostic Autism Spectrum Interview
DAWBA: Development and Well-Being Assessment
DISCO: Diagnostic Interview for Social Communication Disorders
DIVA: Diagnostic Interview of Adult ADHD
DSM: Diagnostic and Statistical Manual of Mental Disorders
EHCP: Educational, Health and Care Plan
FBA: Functional Behavioural Analysis
ICD: International Classification of Diseases
ICT: Information and communication technology
IEP: Individualised Education Programme
K-SADS-PL: Schedule for Affective Disorders and Schizophrenia for School-Age Children-Present and Lifetime Version
LD: Learning disability
NICE: United Kingdom’s National Institute for Health Care Excellence
PEP: Personalised education plan
QbTest: Quantified Behavior Test
R&R: Reasoning and Rehabilitation Programme
SADS: Schedule for Affective Disorders and Schizophrenia
SCD: Social communication disorder
SCDC: Social and Communication Disorders Checklist
SCQ: Social Communication Questionnaire
SDQ: Strengths and Difficulties Questionnaire
SENCO: Special Education Needs Coordinator
SNAP: Swanson, Nolan, and Pelham-IV Questionnaire
SRS: Social Responsiveness Scale
SSRI: Selective serotonin reuptake inhibitor
STD: Sexually transmitted disease
UKAP: United Kingdom ADHD Partnership
WAIS: Wechsler Adult Intelligence Scale
WISC: Wechsler Intelligence Scale for Children

## References

[1] Polanczyk G, De Lima MS, Horta BL, Biederman J, Rohde LA (2007). The worldwide prevalence of ADHD: a systematic review and metaregression analysis. Am J Psychiatry.

[2] Thapar A, Cooper M (2016). Attention deficit hyperactivity disorder. Lancet..

[3] Hollingdale J, Woodhouse E, Young S, Fridman A, Mandy W. Autistic spectrum disorder symptoms in children and adolescents with attention deficit/hyperactivity disorder: a meta-analytical review. Psychol Med, BMC Psychiatry. 19:404. 10.1186/s12888-019-2284-3. 10.1017/S0033291719002368 31530292

[4] Diagnostic and statistical manual of mental disorders. 5th ed (DSM-5). Arlington, VA: American Psychiatric Association. 2013.

[5] Spencer TJ, Biederman J, Mick E (2007). Attention-deficit/hyperactivity disorder: diagnosis, lifespan, comorbidities, and neurobiology. J Pediatr Psychol.

[6] Thomas R., Sanders S., Doust J., Beller E., Glasziou P. (2015). Prevalence of Attention-Deficit/Hyperactivity Disorder: A Systematic Review and Meta-analysis. PEDIATRICS.

[7] Simon V, Czobor P, Bálint S, Mészáros A, Bitter I (2009). Prevalence and correlates of adult attention-deficit hyperactivity disorder: meta-analysis. Br J Psychiatry.

[8] Michielsen M, Semeijn E, Comijs HC, Van de Ven P, Beekman TF, Deeq DJH (2012). Prevalence of attention-deficit hyperactivity disorder in older adults in the Netherlands. Br J Psychiatry.

[9] Faraone SV, Biederman J, Mick E (2006). The age-dependent decline of attention deficit hyperactivity disorder: a meta-analysis of follow-up studies. Psychol Med.

[10] Weiss G, Hechtman LT. Hyperactive children grown up: ADHD in children, adolescents, and adults. 2nd ed. New York: Guilford Press; 1993.

[11] World Health Organization. (2016). International classification of diseases 11th ed (ICD-11)*.* http://www.who.int/classifications/icd/en/. Accessed 21 Apr 2019.

[12] Diagnostic and statistical manual of mental disorders, text revision. 4th ed (DSM-IV). Washington, DC: American Psychiatric Association. 2000.

[13] World Health Organization. (2018). International classification of diseases 11th ed (ICD-11)*.* Update. https://icd.who.int/dev11/f/en#/http%3a%2f%2fid.who.int%2ficd%2fentity%2f821852937. Accessed 21 Apr 2019.

[14] Kooij JJS, Bijlenga D, Salerno L, Jaeschke R, Bitter I, Balázs J (2019). Updated European consensus statement on diagnosis and treatment of adult ADHD. Eur Psychiatry.

[15] Jensen CM, Steinhausen HC (2015). Comorbid mental disorders in children and adolescents with attention-deficit/hyperactivity disorder in a large nationwide study. Atten Defic Hyperact Disord.

[16] Spencer T, Biederman J, Wilens T (1999). Attention-deficit/hyperactivity disorder and comorbidity. Pediatr Clin N Am.

[17] Pliszka SR, Carlson CL, Swanson JM (1999). ADHD with comorbid disorders: clinical assessment and management.

[18] Mancini C, Van Ameringen M, Oakman JM, Figueiredo D (1999). Childhood attention deficit/hyperactivity disorder in adults with anxiety disorders. Psychol Med.

[19] Kessler RC, Adler L, Barkley R, Biederman J, Conners CK, Demler O (2006). The prevalence and correlates of adult ADHD in the United States: results from the National Comorbidity Survey Replication. Am J Psychiatry.

[20] Fayyad J, Sampson NA, Hwang I, Adamowski T, Aquilar-Gaxiola S, Al-Hamzawi A (2017). The descriptive epidemiology of DSM-IV adult ADHD in the World Health Organization World Mental Health Surveys. Atten Defic Hyperact Disord..

[21] Young S, Moss D, Sedgwick O, Fridman M, Hodgkins P (2015). A meta-analysis of the prevalence of attention deficit hyperactivity disorder in incarcerated populations. Psychol Med.

[22] Young S, Sedgwick O, Fridman M, Gudjonsson G, Hodgkins P, Lantigua M (2015). Co-morbid psychiatric disorders among incarcerated ADHD populations: a meta-analysis. Psychol Med.

[23] Elsabbagh M, Divan G, Koh Y-J, Kim YS, Kauchali S, Marcín C (2012). Global prevalence of ASD and other pervasive developmental disorders. Autism Res.

[24] Christensen DL, Maenner MJ, Bilder D, Constantino JN, Daniels J, Durkin MS (2019). Prevalence and characteristics of autism spectrum disorder among children aged 4 years – early autism and developmental disabilities monitoring network, seven sites, United States, 2010, 2012, and 2014. MMWR Surveill Summ.

[25] Murphy CM, Wilson CE, Robertson DM, Ecker C, Daly EM, Hammond N (2016). Autism spectrum disorder in adults: diagnosis, management, and health services development. Neuropsychiatr Dis Treat.

[26] Pourcain BS, Mandy WP, Heron J, Golding J, Smith GD, Skuse DH (2011). Links between co-occurring social-communication and hyperactive-inattentive trait trajectories. J Am Acad Child Adolesc Psychiatry.

[27] Robinson EB, Munir K, Munafò MR, Hughes M, McCormick MC, Koenen KC (2011). Stability of autistic traits in the general population: further evidence for a continuum of impairment. J Am Acad Child Adolesc Psychiatr.

[28] World Health Organization. (2018). WHO releases new International Classification of Diseases (ICD 11). June 18, 2018. https://icd.who.int/browse11/lm/en#/http%3a%2f%2fid.who.int%2ficd%2fentity%2f437815624. Accessed 23 June 2019.

[29] Levy SE, Giarelli E, Lee LC, Schieve LA, Kirby RS, Cunniff C (2010). Autism spectrum disorder and co-occurring developmental, psychiatric, and medical conditions among children in multiple populations of the United States. J Dev Behav Pediatr.

[30] Simonoff E, Pickles A, Charman T, Chandler S, Loucas T, Baird G (2008). Psychiatric disorders in children with autism spectrum disorders: prevalence, comorbidity, and associated factors in a population-derived sample. J Am Acad Child Adolesc Psychiatry.

[31] Hofvander B, Delorme R, Chaste P, Nydén A, Wentz E, Ståhlberg O (2009). Psychiatric and psychosocial problems in adults with normal-intelligence autism spectrum disorders. BMC Psychiatry..

[32] Howlin P, Magiati I (2017). Autism spectrum disorder: outcomes in adulthood. Curr Opin Psychiatry.

[33] Rommelse NN, Franke B, Geurts HM, Hartman CA, Buitelaar JK (2010). Shared heritability of attention-deficit/hyperactivity disorder and autism spectrum disorder. Eur Child Adolescent Psychiatry.

[34] Lee SH, Ripke S, Neale BM, Faraone SV, Purcell SM, Perlis RH (2013). Genetic relationship between five psychiatric disorders estimated from genome-wide SNPs. Nat Genet.

[35] Ronald A, Simonoff E, Kuntsi J, Asherson P, Plomin R (2008). Evidence for overlapping genetic influences on autistic and ADHD behaviours in a community twin sample. J Child Psychol Psychiatry.

[36] Antshel KM, Zhang-James Y, Faraone SV (2013). The comorbidity of ADHD and autism spectrum disorder. Expert Review Neurother.

[37] Jung M, Tu Y, Park J, Jorgenson K, Lang C, Song W (2019). Surface-based shared and distinct resting functional connectivity in attention deficit hyperactivity disorder and autism spectrum disorder. Br J Psychiatry.

[38] Christakou A, Murphy CM, Chantiluke K, Cubillo AI, Smith AB, Giampietro V (2013). Disorder-specific functional abnormalities during sustained attention in youth with attention deficit hyperactivity disorder (ADHD) and with autism. Mol Psychiatry.

[39] Chantiluke K, Christakou A, Murphy CM, Giampietro V, Daly EM, Ecker C (2014). Disorder-specific functional abnormalities during temporal discounting in youth with attention deficit hyperactivity disorder (ADHD), autism and comorbid ADHD and autism. Psychiatry Res.

[40] Boedhoe PSW, van Rooij D, Hoogman M, Twisk JWR, Schmaal L, Abe Y, et al. Subcortical brain volume, regional cortical thickness and surface area variations across attention-deficit/hyperactivity disorder (ADHD), autism spectrum disorder (ASD), and obsessive-compulsive disorder (OCD). In submission. Am J Psychiatry Preprint available doi. 10.1101/673012.

[41] Kern JK, Geier DA, Sykes LK, Geier MR, Deth RC (2015). Are ASD and ADHD a continuum? A comparison of pathophysiological similarities between the disorders. J Atten Disord.

[42] Corbett BA, Constantine LJ, Hendren R, Rocke D, Ozonoff S (2009). Examining executive functioning in children with autism spectrum disorder, attention deficit hyperactivity disorder and typical development. Psychiatry Res.

[43] Leitner Y (2014). The co-occurrence of autism and attention deficit hyperactivity disorder in children – what do we know?. Front Hum Neurosci.

[44] Sinzig J, Walter D, Doepfner M (2009). Attention deficit/hyperactivity disorder in children and adolescents with autism spectrum disorder: symptom or syndrome?. J Atten Disord.

[45] Lee DO, Ousley OY (2007). Attention-deficit hyperactivity disorder symptoms in a clinic sample of children and adolescents with pervasive developmental disorders. J Child Adolescent Psychopharmacol.

[46] Leyfer OT, Folstein SE, Bacalman S, Davis NO, Dinh E, Morgan J (2006). Comorbid psychiatric disorders in children with autism: interview development and rates of disorders. J Autism Dev Disord.

[47] Young S, González R, Mullens H, Mutch L, Mallet-Lambert I, Gudjonsson GH (2018). Neurodevelopmental disorders in prison inmates: comorbidity and combined associations with psychiatric symptoms and behavioural disturbance. Psychiatry Res.

[48] Culpin I, Mars B, Pearson RM, Golding J, Heron J, Bubak I (2018). Autistic traits and suicidal thoughts, plans, and self-harm in late adolescence: population-based cohort study. J Am Acad Child Adolesc Psychiatry.

[49] Hirvikoski T, Mittendorfer-Rutz E, Boman M, Larsson H, Lichtenstein P, Bölte S (2016). Premature mortality in autism spectrum disorder. Br J Psychiatry.

[50] Skirrow C, McLoughlin G, Kuntsi J, Asherson P (2009). Behavioral, neurocognitive and treatment overlap between attention-deficit/hyperactivity disorder and mood instability. Expert Rev Neurother.

[51] Karam RG, Breda V, Picon FA, Rovaris DL, Victor MM, Salgado CAI (2015). Persistence and remission of ADHD during adulthood: a 7-year clinical follow-up study. Psychol Med.

[52] Gershon J (2002). A meta-analytic review of gender differences in ADHD. J Atten Disord.

[53] Lai MC, Lombardo MV, Auyeung B, Chakrabarti B, Baron-Cohen S (2015). Sex/gender differences and autism: setting the scene for future research. J Am Acad Child Adolesc Psychiatry.

[54] Gaub M, Carlson CL (1997). Gender differences in ADHD: a meta-analysis and critical review. J Am Acad Child Adolesc Psychiatry.

[55] Mandy W, Chilvers R, Chowdhury U, Salter G, Seigal A, Skuse D (2012). Sex differences in autism spectrum disorder: evidence from a large sample of children and adolescents. J Autism Dev Disord.

[56] Sun X, Allison C, Auyeung B, Zhang Z, Matthews FE, Baron-Cohen S (2015). Validation of existing diagnosis of autism in mainland China using standardised diagnostic instruments. Autism..

[57] Becker MM, Wagner MB, Bosa CA, Schmidt C, Longo D, Papaleo C (2012). Translation and validation of Autism Diagnostic Interview-Revised (ADI-R) for autism diagnosis in Brazil. Arq Neuropsiquiatr.

[58] Matson JL, Worley JA, Fodstad JC, Chung KM, Suh D, Jhin HK (2011). A multinational study examining the cross-cultural differences in reported symptoms of autism spectrum disorders: Israel, South Korea, the United Kingdom, and the United States of America. Res Autism Spectr Disord.

[59] Sonuga-Barke EJS, Minocha K, Taylor EA, Sandberg S (1993). Inter-ethnic bias in teachers’ ratings of childhood hyperactivity. Br J Dev Psychol.

[60] Larson K, Russ SA, Kahn RS, Halfon N (2011). Patterns of comorbidity, functioning, and service use for US children with ADHD, 2007. Pediatrics..

[61] La Malfa G, Lassi S, Bertelli M, Salvini R, Placidi GF (2004). Autism and intellectual disability: a study of prevalence on a sample of the Italian population. J Intellect Disabil Res.

[62] Wigham S, Rodgers J, Berney T, Le Couteur A, Ingham B, Parr JR (2019). Psychometric properties of questionnaires and diagnostic measures for autism spectrum disorders in adults: a systematic review. Autism..

[63] Rutter M, Bailey A, Lord C. The Social Communication Questionnaire (SCQ). Manual. 2003. Los Angeles: Western Psychological Services.

[64] Constantino JN. Social Responsiveness Scale (SRS™-2), second edition. Manual. 2012.

[65] Skuse DH, Mandy WP, Scourfield J (2005). Measuring autistic traits: heritability, reliability, and validity of the social communication disorders checklist. Br J Psychiatry.

[66] Conners KC. Comprehensive behavior rating scales. Manual. 2008.

[67] Conners KC, Erhardt D, Sparrow E. Conners’ Adult ADHD Rating Scales (CAARS). Manual. 1999.

[68] Swanson JM. The SNAP-IV Teacher and Parent Rating Scale. University of California, Irvine, CA 92715. http://www.shared-care.ca/files/scoring_for_snap_iv_guide_26-item.pdf. Accessed 24 Apr 2020.

[69] Ustun B, Adler LA, Rudin C, Faraone SV, Spencer TJ, Berglund P (2017). The World Health Organization adult attention-deficit/hyperactivity disorder self-report screening scale for DSM-5. JAMA Psychiatry.

[70] Young S. RATE and RATE-C Scales. 2007. https://www.psychology-services.uk.com/adhd.htm. Accessed 24 Apr 2020.

[71] Wolraich M. The Vanderbilt ADHD diagnostic rating scale. https://psychology-tools.com/test/vadrs-vanderbilt-adhd-diagnostic-rating-scale. Assessed 23 June 2019.

[72] Scoring instructions for the NICHQ Vanderbilt Assessment Scales (pdf). American Academy of Pediatrics. https://www.nichq.org/sites/default/files/resource-file/NICHQ_Vanderbilt_Assessment_Scales.pdf. Assessed 23 June 2019.

[73] K-SADS-PL Screener (2016). https://www.kennedykrieger.org/sites/default/files/library/documents/faculty/ksads-dsm-5-screener.pdf. Accessed 21 Apr 2019.

[74] K-SADS-PL DSM-5 Supplement 4: Neurodevelopmental, disruptive and conduct disorders supplement (2016). https://www.pediatricbipolar.pitt.edu/sites/default/files/KSADS_DSM_5_Supp4_DevelopmentalDisruptiveDO_Final.pdf. Accessed 21 Apr 2019.

[75] The Strengths and Difficulties Questionnaire. http://sdqinfo.org/py/sdqinfo/b0.py. Accessed 21 Apr 2019.

[76] Goodman R, Ford T, Richards H, Gatward R, Meltzer H (2000). The development and well-being assessment: description and initial validation of an integrated assessment of child and adolescent psychopathology. J Child Psychol Psychiatry Allied Disciplines.

[77] McEwen Fiona S., Stewart Catherine S., Colvert Emma, Woodhouse Emma, Curran Sarah, Gillan Nicola, Hallett Victoria, Lietz Stephanie, Garnett Tracy, Ronald Angelica, Murphy Declan, Happé Francesca, Bolton Patrick (2015). Diagnosing autism spectrum disorder in community settings using the Development and Well-Being Assessment: validation in a UK population-based twin sample. Journal of Child Psychology and Psychiatry.

[78] Young S. Diagnostic Autism Specftrum Interview. 2020. https://www.psychology-services.uk.com/autism. Accessed 24 Apr 2020.

[79] Rutter M, Le Couteur A, Lord C. ADRI-R: Autism Diagnostic Interview-Revised (ADI-R). 2003. Western Psychological Services, Los Angeles. https://www.scirp.org/(S(351jmbntvnsjt1aadkposzje))/reference/ReferencesPapers.aspx?ReferenceID=1423674 Accessed 21 Apr 2019.

[80] Wing L, Leekam S, Libby S, Gould J, Larcombe M (2002). The diagnostic interview for social and communication disorders: background, inter-rater reliability and clinical use. J Child Psychol Psychiatry.

[81] Skuse D, Warrington R, Bishop D, Chowdhury U, Lau J, Mandy W (2004). The developmental, dimensional and diagnostic interview (3Di): a novel computerized assessment for autism spectrum disorders. J Am Acad Child Adolesc Psychiatry.

[82] Young S. ACE: ADHD Child Interview. 2015. https://www.psychology-services.uk.com/adhd. Accessed 24 Apr 2020.

[83] Young S. ACE+: ADHD Interview Adult. 2016. https://www.psychology-services.uk.com/adhd. Accessed 24 Apr 2020.

[84] Kooij JJ (2013). Adult ADHD: diagnostic assessment and treatment.

[85] Furczyk K, Thorne J (2014). Adult ADHD and suicide. Atten Defic Hyperact Disord.

[86] Veenstra-VanderWeele J (2018). Recognizing the problem of suicidality in autism spectrum disorder. J Am Acad Child Adolesc Psychiatry.

[87] Lord C, Luyster RJ, Gotham K, Guthrie W (2012). Autism diagnostic observation schedule, second edition (ADOS-2) manual (part II): toddler module.

[88] Lord C, Rutter M, DiLavore PC, Risi S, Gotham K, Bishop S (2012). Autism diagnostic observation schedule, second edition (ADOS-2) manual (part I): modules 1–4.

[89] Wechsler D (2014). Wechsler intelligence scale for children-fifth edition.

[90] Wechsler D (2008). Wechsler adult intelligence scale - fourth edition.

[91] Harrison P, Oakland T (2015). Adaptive behavior assessment (ABAS-3) – third edition.

[92] Sparrow SS, Cicchetti DV, Saulnier CA (2016). Vineland adaptive behavior scales (Vineland-3) - third edition.

[93] Kaufman AS, Kaufman NL (2018). Kaufman assessment battery for children. Second edition. Normative update.

[94] Conners CK (2014). Conners’ continuous performance test - third edition.

[95] Ulberstad F (2012). QbTest technical manual.

[96] Young S, Amarasinghe JA (2010). Practitioner review: non-pharmacological treatments for ADHD: a lifespan approach. J Child Psychol Psychiatry.

[97] Pickles A, Le Couteur A, Leadbitter K, Salomone E, Cole-Fletcher R, Tobin H (2016). Parent-mediated social communication therapy for young children with autism (PACT): long-term follow-up of a randomized controlled trial. Lancet..

[98] Green J, Charman T, McConachie H, Aldred C, Slonims V, Howlin P (2010). Parent-mediated communication-focused treatment in children with autism (PACT): a randomized controlled trial. Lancet..

[99] Kasan C, Gulsrud A, Paparella T, Hellemann G, Berry K (2015). Randomized comparative efficacy study of parent-mediated interventions for toddlers with autism. J Consult Clin Psychol.

[100] Smith T, Iadarola S (2015). Evidence base update for autism spectrum disorder. J Clin Child Adolesc Psychol.

[101] Young Susan, Smith Jade (2017). Helping Children with ADHD.

[102] Young S (2017). The STAR detective facilitator manual: a cognitive behavioral group intervention to develop skilled thinking and reasoning for children with cognitive, behavioral, emotional and social problems.

[103] Young S (2017). Becoming a STAR detective!: your detective’s notebook for finding clues to how you feel.

[104] Marshall D, Thomas T (2015). Polygraphs and sex offenders: the truth is out there. Probation J.

[105] Gudjonsson GH, Vrij A. A review of the current scientific status and fields of application of polygraphic deception of detection. Leicester: British Psychological Society; 2004. p. 1–31.

[106] Pfiffner LJ, Rooney M, Haack L, Villodas M, Delucchi K, McBurnett K (2016). A randomized controlled trial of a school-implemented school-home intervention for attention-deficit/hyperactivity disorder symptoms and impairment. J Am Acad Child Adolesc Psychiatry.

[107] Pfiffner LJ, Rooney ME, Jiang Y, Haack LM, Beaulieu A, McBurnett K (2018). Sustained effects of collaborative school-home intervention for attention-deficit/hyperactivity disorder symptoms and impairment. J Am Acad Child Adolesc Psychiatry.

[108] Shaw P, Eckstrand K, Sharp W, Blumenthal J, Lerch JP, Greenstein D (2007). Attention-deficit/hyperactivity disorder is characterized by a delay in cortical maturation. Proc Natl Acad Sci U S A.

[109] The Joint Council for Qualifications. https://www.jcq.org.uk/. Accessed 28 Apr 2019.

[110] The Scottish Qualifications Authority. https://www.sqa.org.uk/sqa/70972.html. Accessed 28 Apr 2019.

[111] Harpin VA (2005). The effect of ADHD on the life of an individual, their family, and community from preschool to adult life. Arch Dis Child.

[112] Østergaard SD, Dalsgaard S, Faraone SV, Munk-Olsen T, Laursen TM (2017). Teenage parenthood and birth rates for individuals with and without attention-deficit/hyperactivity disorder: a nationwide cohort study. J Am Acad Child Adolesc Psychiatry.

[113] Emilsson, Gudjonsson G, Sigurdsson JF, Einarsson E, Baldursson G, Olafsdottir H (2011). Cognitive behaviour therapy in medication-treated adults with ADHD and persistent symptoms: a randomized controlled trial. BMC Psychiatry.

[114] Young S, Khondoker M, Emilsson B, Sigurdsson JF, Philipp-Wiegmann F, Baldursson G (2017). A randomized controlled trial reporting functional outcomes of cognitive behavioural therapy in medication-treated adults with ADHD and comorbid psychopathology. Eur Arch Psychiatry Clin Neurosci.

[115] Young S, Khondoker M, Emilsson B, Sigurdsson JF, Philipp-Wiegmann F, Baldursson G, Olafsdottir H, Gudjonsson G (2015). Cognitive behavioral therapy in medication-treated adults with ADHD and comorbid psychopathology: a randomized controlled trial using multi-level analysis. Psychol Med.

[116] Waugh A, Gudjonsson GH, Rees-Jones A, Young S (2014). A feasibility study of the Reasoning and Rehabilitation Mental Health Programme (R&R2MHP) in male offenders with intellectual disability. Criminal Behaviour Mental Health. 2014; (24)222–4. 10.1002/cbm.1907 25042837

[117] Bramham J, Young S, Bickerdike Spain D, McCartan D, Xenitidis K (2009). Evaluation of group cognitive behavioral therapy for adults with ADHD. J Atten Disord.

[118] Knouse LE, Teller J, Brooks MA (2017). Meta-analysis of cognitive–behavioral treatments for adult ADHD. J Consult Clin Psychol.

[119] Spain D, Sin J, Chalder T, Murphy D, Happe F (2015). Cognitive behaviour therapy for adults with autism spectrum disorders and psychiatric co-morbidity: a review. Res Autism Spectr Disord.

[120] Solanto MV, Marks DJ, Wasserstein J, Mitchell K, Abikoff H, Alvir JM (2010). Efficacy of meta-cognitive therapy for adult ADHD. Am J Psychiatry.

[121] Russell AJ, Jassi A, Fullana MA, Mack H, Johnston K, Heyman I (2013). Cognitive behavior therapy for comorbid obsessive-compulsive disorder in high-functioning autism spectrum disorders: a randomized controlled trial. Depress Anxiety.

[122] Russell AJ, Mataix-Cols D, Anson MA, Murphy DG (2009). Psychological treatment for obsessive-compulsive disorder in people with autism spectrum disorders – a pilot study. Psychother Psychosom.

[123] Young S, Adamou M, Asherson P, Coghill D, Colley B, Gudjonsson G (2016). Recommendations for the transition of patients with ADHD from child to adult healthcare services: a consensus statement from the UK Adult ADHD Network. BMC Psychiatry..

[124] Young S, Murphy C, Coghill D (2011). Avoiding the ‘twilight zone’: guidance and recommendations on ADHD and the transition between child and adult services. BMC Psychiatry.

[125] Civil Service Judgement Test: A guide for candidates. Accessed 07 May 2019. https://www.google.com/search?client=safari&rls=en&q=Civil+Service+Situational+Judgment+Test&ie=UTF-8&oe=UTF-8.

[126] Sturman N, Deckx L, van Driel ML. Methylphenidate for children and adolescents with autism spectrum disorder. Cochrane Database Syst Rev. 2017; 10.1002/14651858.CD011144.pub2. 10.1002/14651858.CD011144.pub2 PMC6486133 29159857

[127] Reichow B, Volkmar FR, Bloch MH (2013). Systematic review and meta-analysis of pharmacological treatment of the symptoms of attention-deficit/hyperactivity disorder in children with pervasive developmental disorders. J Autism Dev Disord.

[128] Howes OD, Rogdaki M, Findon JL, Wichers RH, Charman T, King BH (2018). Autism spectrum disorder: consensus guidelines on assessment, treatment and research from the British Association for Psychopharmacology. J Psychopharmacol.

[129] Taylor DM, Barnes TR, Young AH. The Maudsley prescribing guidelines in psychiatry, 13th edition. Chichester: Wiley; 2018.

[130] Reiersen AM, Handen B (2011). Commentary on ‘selective serotonin reuptake inhibitors (SSRIs) for autism spectrum disorders (ASD)’. Evid Based Child Health.

[131] Santosh PJ, Baird G, Pityaratstian N, Tavare E, Gringras P (2006). Impact of comorbid autism spectrum disorders on stimulant response in children with attention deficit hyperactivity disorder: a retrospective and prospective effectiveness study. Child Care Health Dev.

[132] Politte LC, Scahill L, Figuera J, McCracken JT, King B, McDougle CJ (2018). A randomized, placebo-controlled trial of extended-release guanfacine in childrenwith autism spectrum disorder and ADHD symptoms: an analysis of secondary outcome measures. Neuropsychopharmacol.

[133] Handen BL, Aman MG, Arnold LE, Hyman SL, Tumuluru RV, Lecavalier L (2015). Atomoxetine, parent training, and their combination in children with autism spectrum disorder and attention-deficit/hyperactivity disorder. J Am Acad ChildAdolesc Psychiatry.

[134] Friedland S, Walkup JT (2015). Meta-assurance: no tic exacerbation caused by stimulants. J Am Acad Child Adolesc Psychiatry.

